# 2D Layered Double Hydroxide Nanosheets and Their Derivatives Toward Efficient Oxygen Evolution Reaction

**DOI:** 10.1007/s40820-020-00421-5

**Published:** 2020-04-06

**Authors:** Xueyi Lu, Hairong Xue, Hao Gong, Mingjun Bai, Daiming Tang, Renzhi Ma, Takayoshi Sasaki

**Affiliations:** International Center for Materials Nanoarchitectonics (WPI-MANA), National Institute for Materials Science (NIMS), Tsukuba, Japan

**Keywords:** Layered double hydroxides, Nanosheets, Derivatives, Catalysts, Oxygen evolution reaction

## Abstract

Layered double hydroxides (LDHs) have attracted tremendous research interest in widely spreading applications. Most notably, transition-metal-bearing LDHs are expected to serve as highly active electrocatalysts for oxygen evolution reaction (OER) due to their layered structure combined with versatile compositions. Furthermore, reducing the thickness of platelet LDH crystals to nanometer or even molecular scale via cleavage or delamination provides an important clue to enhance the activity. In this review, recent progresses on rational design of LDH nanosheets are reviewed, including direct synthesis via traditional coprecipitation, homogeneous precipitation, and newly developed topochemical oxidation as well as chemical exfoliation of parent LDH crystals. In addition, diverse strategies are introduced to modulate their electrochemical activity by tuning the composition of host metal cations and intercalated counter-anions, and incorporating dopants, cavities, and single atoms. In particular, hybridizing LDHs with conductive components or in situ growing them on conductive substrates to produce freestanding electrodes can further enhance their intrinsic catalytic activity. A brief discussion on future research directions and prospects is also summarized.

## Highlights

- Synthesis strategies of layered double hydroxides (LDHs) were summarized with classifications of traditional coprecipitation, homogeneous precipitation, and newly developed topochemical oxidation.
- Diverse approaches of structural modulation and hybridization to enhance the electrocatalytic activity of LDHs were systematically reviewed.

## Graphical abstract

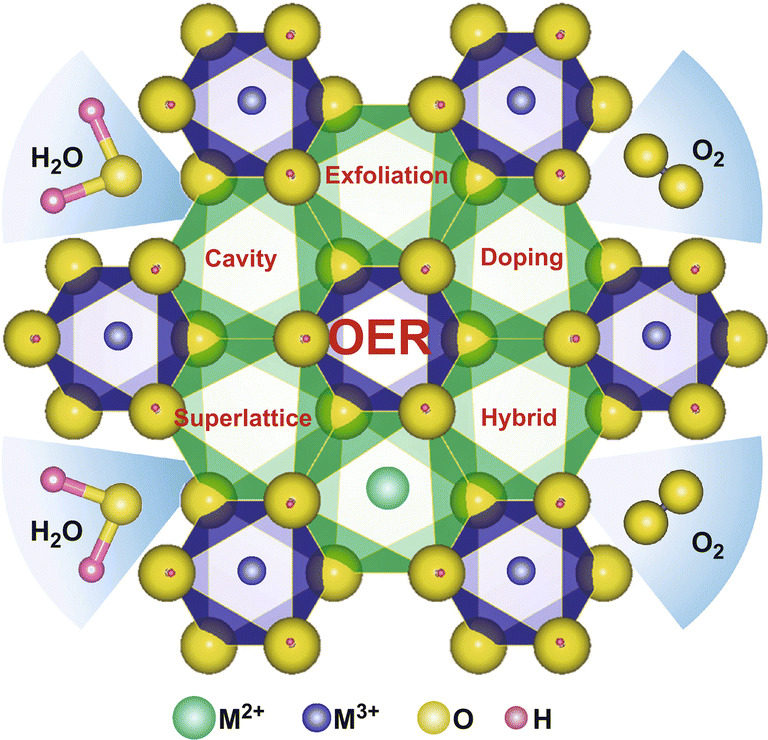

## Introduction

Electrochemical water splitting, involving hydrogen evolution reaction (HER) and oxygen evolution reaction (OER), is considered as one of alternative renewable energy systems to replace traditional fossil fuels [1–3]. Compared with other alternatives such as solar and wind power, water splitting is more efficient to utilize intermittent energy by converting electricity to clean chemical energy carriers (e.g., hydrogen) [4]. However, both reactions of water splitting suffer from sluggish kinetics due to the inertia, especially for the OER which experiences a four-electron (4e) transfer process [5]. Exploring efficient electrocatalysts is crucial to address this issue. The state-of-the-art commercial catalysts of water splitting rely on precious metals of the platinum group, with Ru, Ir, and their oxides toward OER, while Pt is the benchmark catalyst for HER [6, 7]. However, the scarcity and precious nature hinder their large-scale application. Moreover, Ru, Ir, and their oxides might suffer from oxidation at high potential and Pt would undergo dissolution in the electrolyte during long-time operation [8–10].

**Fig. 1:**
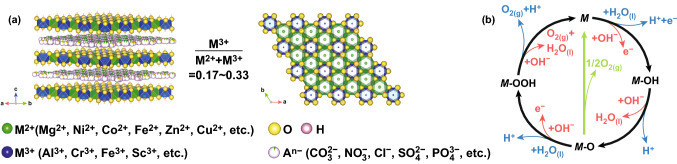
**a** Typical structure model of LDHs and in-plane cation arrangement. **b** OER mechanisms in acid (blue) and alkaline (red) conditions. Reproduced with permission from Ref. [23]. Copyright 2006 The Royal Society of Chemistry

During the past decades, substantial efforts have been devoted to developing high-performance catalysts based on low-cost transition metal elements. Among them, layered double hydroxides (LDHs) recently attracted considerable research interest owing to their intriguing electrocatalytic activities, earth abundance, ultrastability, and low-toxic properties [11–14]. LDHs can be represented by a general formula as M_1−*x*_^2+^M_*x*_^3+^(OH)_2_(A^*n*−^)_*x/n*_·*y*H_2_O [15–21]. As depicted in Fig. 1, LDHs consist of brucite-like layers with a fraction of octahedrally coordinated divalent metal cations replaced by trivalent ones, resulting in positive charge of the host layers. Exchangeable inorganic or organic anions are accommodated in the interlayer galleries to compensate for the positive charge. Moreover, the hydroxyl groups of the host layers are connected to the anions or water molecules by hydrogen bonds. The *x* value equals the molar ratio of M^3+^/(M^2+^+M^3+^), which is in the range of 0.17–0.33 [22]. Thanks to the controllable M^2+^/M^3+^ molar ratio, tunability of the metal cations, and exchangeable charge-compensating anions, a large number of host–guest assemblies and nanoarchitectures can contribute to the design for desirable physical and chemical properties. The properties of LDHs have been tailored to fulfill the specific demand of different applications, ranging from electrocatalysis, photoelectrocatalysis, adsorption materials to additives in polymers, and so on. During the OER catalytic process, both M^2+^ and M^3+^ are involved in redox reactions by adsorbing and desorbing reactants, intermediates, and products (Fig. 1b) [23]. There may be possible electron transfer occurring between M^2+^ and M^3+^ in the process. A^n−^, acting as the counterions to compensate for the positive charge, may affect adsorption/desorption process on LDH host layer surface and/or in the interlayer space.

The lateral size and thickness have a great effect on physical and chemical properties [24]. In particular, when the thickness reduced to nanometer or even molecular scale, the resulting nanosheets can maximize the exposing active sites and specific surface area, which would effectively contribute to increased intrinsic electrocatalytic activities, facilitate the transport of reactant species, and finally promote the activity toward water splitting [25, 26].

In this review, we summarize recent progress in the structure design and exfoliation of LDHs, development of their derivates as well as exploring their applications in electrochemical water splitting. First, we will introduce various preparation methods of LDH nanosheets, mainly classified into two categories—direct synthesis in a bottom-up approach and top-down chemical exfoliation of the layered precursory compounds. Aiming to overcome the poor conductivity of LDHs, various strategies will be covered, including doping metal or nonmetal elements, introducing cavities, decorating LDHs with functional nanoparticles, hybridizing LDHs with conductive components, and growing LDHs on conductive substrate to produce 3D freestanding electrodes. Based on these developments, we highlight the applications of LDHs and their derivatives as OER catalysts toward electrochemical water splitting.

## Synthetic Strategies

The reliable synthesis of 2D LDHs with controllable lateral size and thickness is crucial for exploring their structural, physical, and electrochemical properties. Stimulated by their interesting potential applications, tremendous efforts have been devoted to developing various strategies for producing 2D LDHs. The typical preparation methods of 2D LDHs can be classified into “top-down” and “bottom-up” approaches. The general “top-down” approach usually uses physical shear force or chemical intercalation to break the interaction between adjacent layers, attaining mono- or few-layered nanosheets from their bulk counterparts. Oppositely, the bottom-up method relies on the direct preparation of 2D LDH nanosheets via chemical reactions/syntheses.

### Direct Synthesis of LDH Nanosheets

Direct synthesis of 2D nanosheets may be considered as a straightforward and attractive bottom-up procedure [15]. Various mechanical and chemical strategies toward direct synthesis of LDH nanosheets have been explored, including applying a laser beam on metals in aqueous solution, utilizing a layer growth inhibitor in a microemulsion, and using a special reactor to create a rapid reaction environment, etc.

#### Physicochemical Approach

##### Pulsed Laser Ablation

**Fig. 2:**
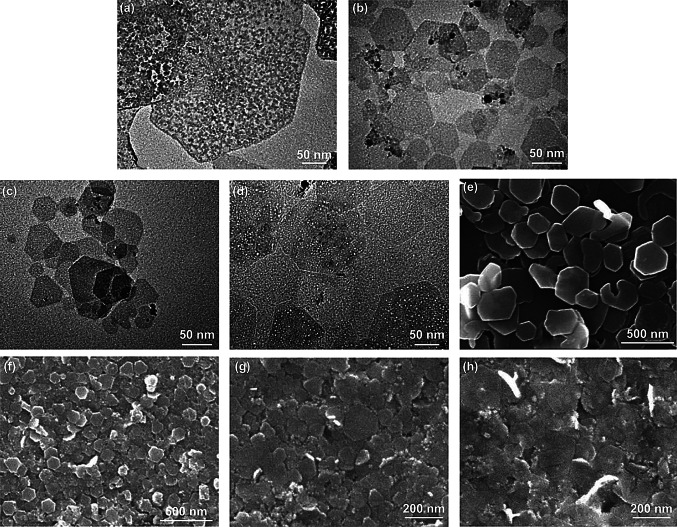
TEM and SEM images of four different LDH nanosheets prepared by pulsed laser ablation. **a**, **e** Zn–Al LDH, **b**, **f** Co–Fe LDH, **c**, **g** Co–Al LDH, **d**, **h** Mg–Fe LDH. Reproduced permission from Ref. [27]. Copyright 2010 American Institute of Physics

Hur et al. [27] introduced a new method to synthesize LDHs and their ultrathin nanosheets in de-ionized water by pulsed laser ablation without any chemical or heat treatment. Laser ablation technique has recently been used for the formation of nanomaterials in liquid environment, including Ag particles [28], ZnO particles [29], Au–Ag alloy [30, 31], and other stable phases depending on the properties of target materials and surrounding liquid. The preparation process of LDHs was carried out in two steps. The first is laser ablation of a metal target for trivalent cations in de-ionized water at room temperature using Q-switched Nd–yttrium aluminum garnet laser, and the second is laser ablation over another metal target for bivalent source in the previously prepared trivalent metallic colloid. By controlling the ablation time, wavelength, and fluence, Zn–Al, Co–Fe, Co–Al, and Mg–Fe LDHs were formed with a molecular thickness, corresponding to the thickness of the exfoliated 2D nanosheets (Fig. 2a–d). Figure 2e–h shows that the lateral sizes of these LDHs are approximately 300, 100, 100, and 200 nm, respectively. Moreover, all these colloidal nanosheets were found to be stable without any agglomeration or formation of lamellar structures. Among them, Mg–Fe and Co–Fe LDH layers present extremely large lateral size comparable with that of the regular LDHs when being prepared quickly on transmission electron microscopy (TEM) grid. The TEM images showed that the Mg–Fe LDHs present a rolling and folding morphology with a thickness approaching 0.5 nm.

Employing the method of pulsed laser ablation in liquid (PLAL), Müller et al. synthesized a series of [Ni–Fe]-LDHs with intercalated nitrate ions and water—[Ni_1−*x*_Fe_*x*_(OH)_2_](NO_3_)_*y*_(OH)_*x*–*y*_·*n*H_2_O [32]. Iron or nickel powder was firstly mixed in 10 mL aqueous metal nitrate solutions using a magnetic stirrer. For the formation of bimetallic LDHs, one kind of metal was used as the ablation target, while the nitrate salt of the other metal was dissolved in the precursor solution. During PLAL, nanoparticles were formed by rapid cooling of plasma comprised of elements for the solid ablation target and the surrounding liquid. After the synthesis process, the LDH nanoparticle suspensions were separated from the metallic ablation targets using a strong magnet. The composition of LDHs with mixed metals was carefully controlled by varying the ablation targets, type of metal ions, and their concentrations, as well as laser pulse energies. Powder X-ray diffraction (XRD) measurements implied that the Fe-rich nanoparticles are poorly crystalline, while the Ni-rich nanoparticles display diffraction patterns consistent with the LDH structure. Mössbauer and X-ray absorption spectroscopic data indicated that the Fe was incorporated as Fe^3+^ to replace partial Ni^2+^ in [Ni–Fe]-LDHs. The TEM data showed that the lateral sizes ranged from ~ 7 to 22 nm.

Laser ablation process has proved to be an attractive method to prepare ultrathin LDH layers with a uniform size. More importantly, the high laser power and short reaction time can effectively alleviate the contamination of carbonates.

##### Microwave Irradiation

**Fig. 3:**
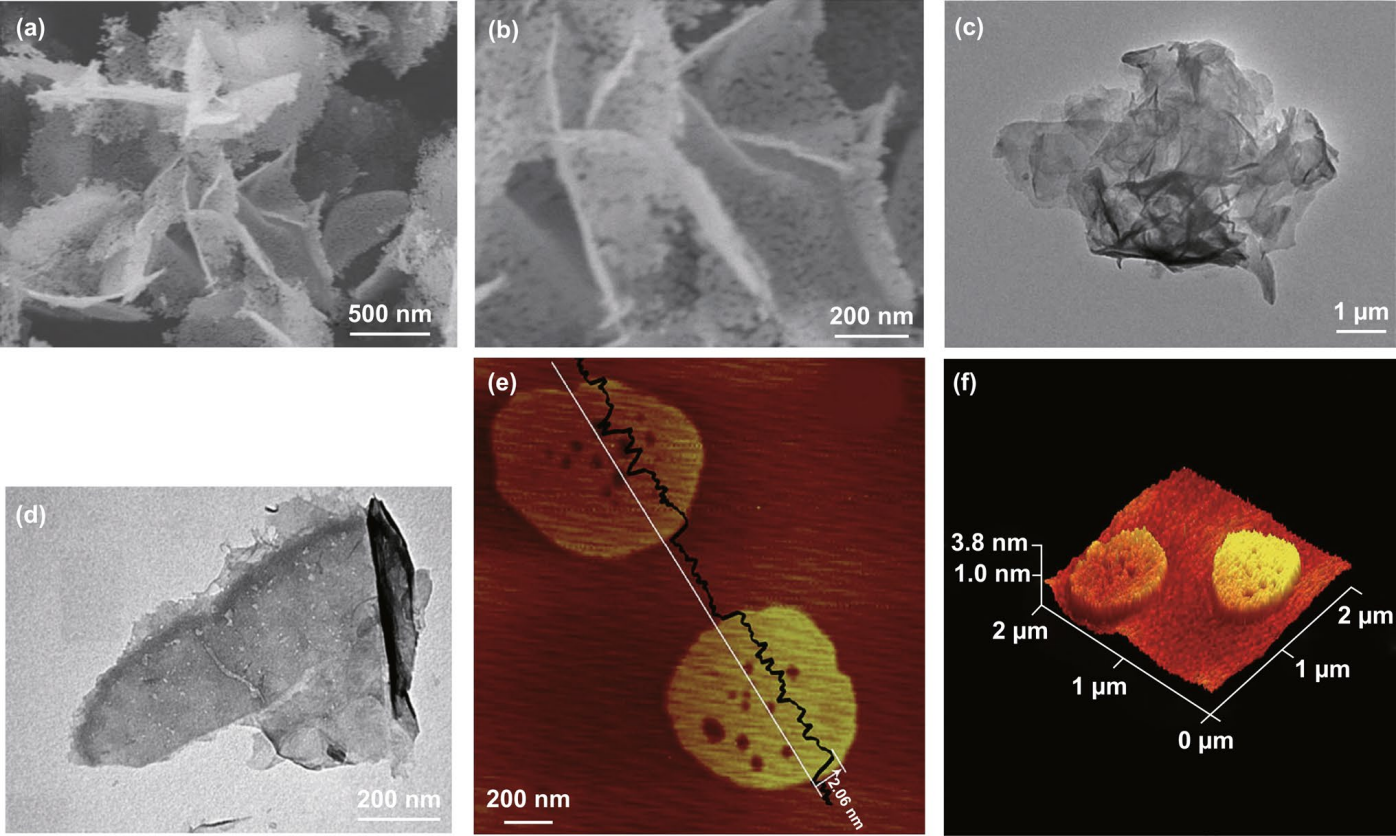
**a**, **b** SEM and **c**, **d** TEM images of Zn–Co-LDH nanosheets. **e** Height profile and **f** the 3D PF-AFM images of Zn–Co-LDH nanosheets prepared by microwave irradiation. Reproduced with permission from Ref. [33]. Copyright 2015 The Royal Society of Chemistry

Microwave irradiation serves as a facile and convenient way to synthesize uniform materials by altering the reaction kinetics and selectivity during the nucleation process. Xu et al. used a one-step microwave-assisted approach to prepare Zn–Co-LDH nanosheets, which avoided the low yield and the complex synthesis via the liquid exfoliation method (Fig. 3a–d) [33]. Zinc nitrate hexahydrate, cobalt nitrate hexahydrate, and urea were firstly dissolved in de-ionized water and then transferred to a round-bottomed flask, followed by microwave irradiation at 900 W. They found that the XRD peaks of Zn–Co-LDHs, especially the 003, 006, and 113 peaks, became stronger with the increase in microwave power. The AFM images demonstrated that the reaction time had a large effect on the morphology, the lateral size getting larger with increasing the reaction time. The thickness of the obtained nanosheets was measured to be ~ 2 nm by peak-force-model atomic force microscopy (PF-AFM) (Fig. 3e, f). Such a simple and effective method could be extended to large-scale synthesis.

#### Chemical Approach

**Fig. 4:**
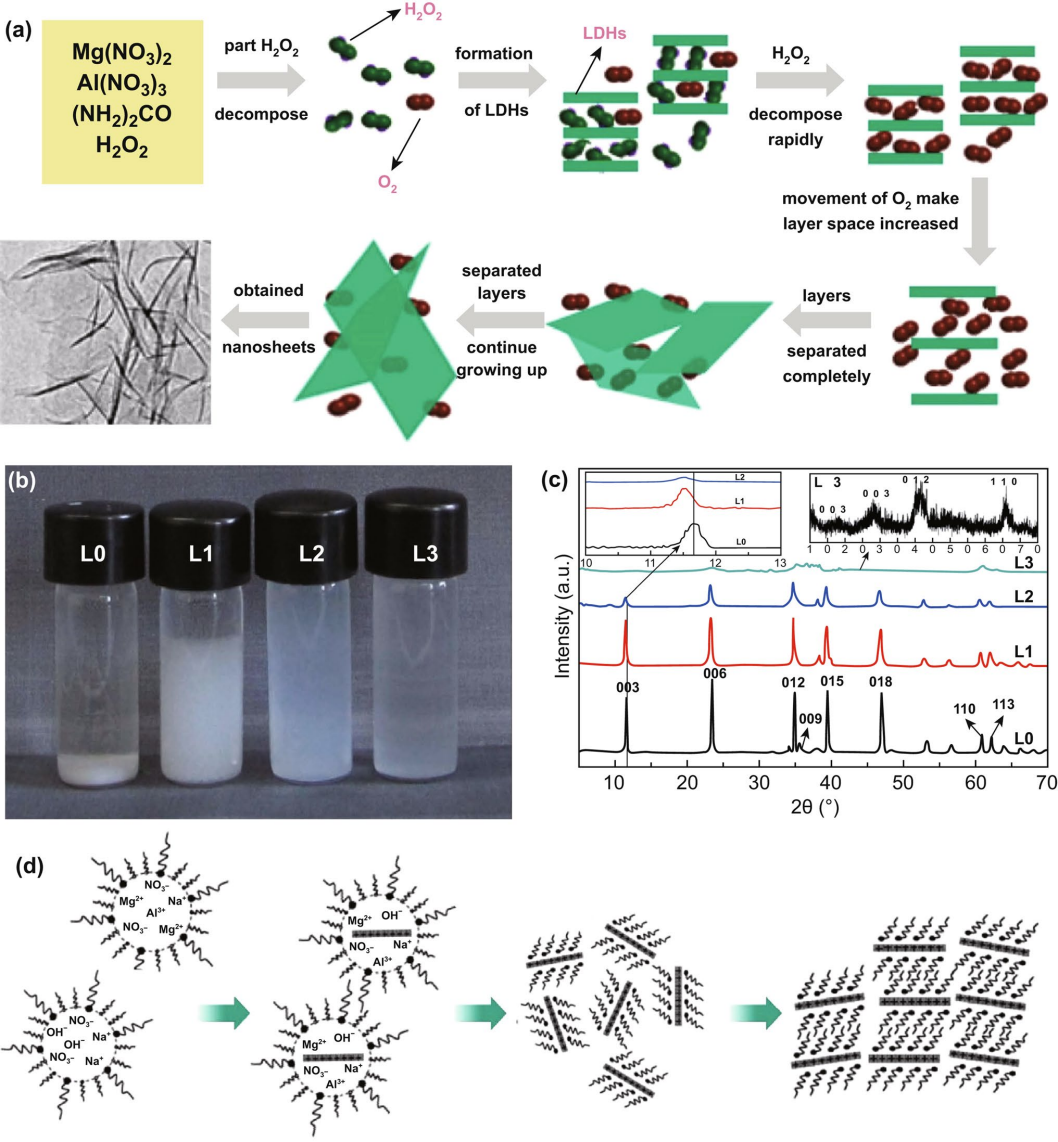
**a** Suggested scheme for preparing exfoliated MgAl-LDH nanosheets. **b**, **c** Digital graphs of MgAl-LDHs suspension with different contents of H_2_O_2_ and the corresponding XRD patterns. Reproduced with permission from Ref. [34]. Copyright 2012 Elsevier Inc. **d** Schematic illustration of the nucleation and growth of LDH platelets. Reproduced with permission from Ref. [35]. Copyright 2006 The Royal Society of Chemistry

Yan et al. developed a single-step method to synthesize MgAl-LDHs ultrathin nanosheets in large scale with the assistance of hydrogen peroxide (Fig. 4a) [34]. Mg(NO_3_)_2_·6H_2_O, Al(NO_3_)_3_·9H_2_O, and urea were firstly dissolved into 100 mL 30% H_2_O_2_ to yield a solution containing 0.01 M Mg^2+^ and 0.005 M Al^3+^. Then, the mixture was loaded into a Teflon tube and heated at 150 °C for 24 h. A translucent colloidal suspension was achieved after the reaction. The key point to obtain LDH nanosheets is that the oxygen molecules derived from in situ decomposition of H_2_O_2_ are accommodated in the interlayer space of the resulting LDHs. Due to their violent movements, the interlayer spacing increased and the electrostatic interaction of layers diminished until layers separated completely. It is illustrated that as the percentage of H_2_O_2_ increases, the resultant solution becomes more transparent with higher yield (Fig. 4b). The 003 XRD peak shifted to a low angle when adding H_2_O_2_, indicating that the interlayer spacing of MgAl-LDHs was expanded. On reaching 30% H_2_O_2_, a semitransparent colloidal suspension was obtained which could be kept stable for several weeks in air without the formation of precipitates. SEM image exhibited hexagonal morphology of the MgAl-LDHs plates with a size of 5–10 μm. The height analysis was carried out by AFM at steps between a nanosheet and the substrate surface, yielding a thickness value of 1.44 nm.

Hu et al. reported a facile one-step synthesis of LDH monolayers in a reverse microemulsion (Fig. 4d) [35]. Mg(NO_3_)_2_·6H_2_O and Al(NO_3_)_3_·9H_2_O were introduced into an oil phase of isooctane with sodium dodecyl sulfate as surfactant and 1-butanol as co-surfactant. The pH of the solution was adjusted to 10 by NaOH. The aqueous phase containing the nutrients for the growth of LDH would be dispersed in the oil phase to form droplets surrounded by dodecyl sulfate groups. The droplets served as nanoreactors and provided limited space and nutrients for the formation of LDH platelets. Therefore, both the diameter and thickness can be effectively controlled. Such system also allowed the negatively charged dodecyl sulfate chains to interact with the LDH planes to balance the charge. The XRD patterns of the gel-like materials presented two broad reflections at ca. 2*θ* = 7.5° and 20°, while strong basal plane Bragg reflection of LDHs was missing, suggesting a lack of an organized layered structure of the sample. Upon drying, the pattern showed a gradual growth of a 2*θ* = 3° Bragg reflection, indicating that the sample gains some structural order. All those reflections could be indexed as a rhombohedral unit cell with *a *= 2*d*_110_ = 3.04 Å and *c *= 3*d*_003_ = 77.88 Å, agreeing well with the unit cell dimensions of the Mg_2_Al-LDH intercalated with dodecyl sulfate that was obtained by ion exchange method from the pristine nitrate form. The AFM topology revealed isolated oval objects which have a uniform height distribution around 1.5 nm and a diameter distribution centered around 40 nm.

### Chemical Exfoliation of Parent LDH Crystals

Chemical exfoliation of parent LDH crystals provides another effective route to produce 2D nanosheets. Compared with direct synthesis process, it is more facile to control the growth rate and chemical composition of LDHs. Moreover, ion intercalation/exchange is a crucial step in chemical exfoliation route, which introduces diverse anions into the interlayer space of LDHs, contributing to the modification of LDH structure and tuning of properties.

#### Synthesis of Layered Precursor Compounds

The prerequisite of top-down strategy is to obtain multilayered host compounds in high quality. LDH crystals are usually prepared via a solution-based process, which may be classified as coprecipitation, homogeneous precipitation, and topochemical oxidation. Coprecipitation involves the precipitation of a solution containing divalent and trivalent metal salts under an alkaline condition or constant pH, e.g., by adding NaOH or Na_2_CO_3_ [36–38]. In the early stages, almost all of the LDH parent materials were prepared by the coprecipitation. Later, homogeneous precipitation typically uses a reagent such as urea (CO(NH_2_)_2_ or hexamethylenetetramine (HMT, C_6_H_12_N_4_), which is hydrolyzed to slowly release ammonia and to generate an alkaline environment. Compared with coprecipitation, homogeneous precipitation generally leads to LDH products in high crystallinity due to homogenous nucleation and growth procedure [39, 40]. On the other hand, topochemical oxidation, a newly developed process involving a topotactic oxidative intercalation, started from brucite-like divalent metal hydroxides [41]. Using cobalt chloride (CoCl_2_·6H_2_O) and ferrous chloride (FeCl_2_·4H_2_O) as the precursors, Ma et al. [42] developed a new process to synthesize transition-metal-bearing LDHs; brucite-like Co^2+^–Fe^2+^ hydroxide was firstly synthesized via HMT hydrolysis under nitrogen protection, and the product was later transformed to Co_2/3_Fe_1/2_(OH)_2_ LDH via topotactic oxidative intercalation with iodine in chloroform (I_2_/CHCl_3_). Brownish product was obtained by filtering and rinsing with anhydrous ethanol repeatedly. This innovative topochemical approach was also employed to successfully prepare Co^2+^–Co^3+^ LDH, which cannot be produced by traditional coprecipitation or homogeneous precipitation method because there is no stable dissociated Co^3+^ or Co(OH)_3_ in the aqueous solution [43]. Different from the heterometallic (Co–Fe) hydroxide, more crucial control was required to complete the oxidation degree of the same element by incorporating mixed valences. For example, after achieving the brucite-type hydroxide of hexagonal β-Co(OH)_2_ by refluxing CoCl_2_ in HMT solution, Ma et al. [43] employed bromine (Br_2_) in acetonitrile (CH_3_CN) to transform it into Co^2+^–Co^3+^ LDH. They found that the oxidative intercalation process consumed 40 times the required amount of Br_2_ and the oxidation took 5 days to ensure the complete conversion into single Co^2+^–Co^3+^ LDH phase without residue. Lee et al. developed a novel synthetic approach to hydrotalcite-like Co^2+^ (or Ni^2+^)–Fe^3+^-LDHs using a one-pot topochemical oxidation reaction by anthraquinone-2-sulfonate anions (AQS2) [44]. AQS2 served as a mild oxidizing agent which allows the sole oxidation of Fe^2+^ into Fe^3+^ to form the LDH phase and were intercalated into the interlayer space of the LDH during the course of a slow precipitation. The process was also carried out by refluxing the CoCl_2_ (or NiCl_2_)–FeCl_2_–AQS2–HMT solution with a stoichiometric ratio 2:1 of Co (or Ni)/Fe under N_2_ atmosphere for 3 h. The standard redox potential of AQS2^2−^/AQS2 was − 0.6 V (vs. Ag/AgCl), which is possible to oxidize the Fe^2+^ ions in the Fe(OH)_2_ phase because of the lower standard potential in Fe(OH)_3_/Fe(OH)_2_ (− 0.58 V). Brown and dark yellow solid precipitates were attained for the Co–Fe LDH and Ni–Fe LDH, respectively. The average lateral size of both samples was detected to be 0.5 μm, and the thickness was around 70 nm. All diffraction peaks of XRD patterns were readily indexed as a hydrotalcite-like phase similar to those in α-Co-AQS2-LDH phase [45].

#### Ion Intercalation/Exchange

Ion intercalation/exchange is effective to synthesize LDH with desired ions and in turn regulate the expected properties of materials [46–49]. Ion intercalation spontaneously happens during the chemical synthesis of LDHs because counterions are needed to balance the host layer charge. In addition to inorganic anions including alkoxide, molybdate, polyoxometalates, etc., various organic molecules, such as glucose [50], carbon dots [51], and ethylene glycol [52], can also be intercalated. Intercalating guests between LDH host layers not only expand the interlayer spacing for convenient transport of ions/electrons, but also facilitate the subsequent exfoliation of LDHs into ultrathin nanosheets [53].

#### Exfoliation

Bulk LDHs are solids with strong in-plane chemical bonds while relatively weak interlayer forces. Exfoliating or delaminating bulk LDHs into few-layered or monolayered nanosheets could expose abundant active sites and contribute to an enhanced intrinsic catalytic activity. Since the report of exfoliation of graphite into monolayer graphene, diverse approaches, including soft chemical exfoliation and plasma exfoliation, have been developed and widely used.

##### Soft Chemical Exfoliation in Liquid/Solvent

The first attempt to exfoliate LDH was reported by Adachi-Pagano et al. [54]. They prepared dodecyl sulfate (DS^−^, C_12_H_25_SO_4_^−^)-intercalated Zn_2_/Al–DS^−^−LDH and tried delaminating it in various organic solvents. The results showed that the LDH could be exfoliated in butanol, pentanol, and hexanol and remain stable for a long time. Zn_2_/Al–DS^−^−LDH was partially delaminated in other solvents, such as water, methanol, ethanol, propanol, and hexane. Hydration state of the DS^−^−LDH was found to be a vital factor for determining the exfoliation extent. Venugopal et al. applied such exfoliation method to different types of divalent and trivalent LDHs, including Mg/Al-LDHs, Ni/Al-LDHs, and Zn/Al-LDHs intercalated with sodium dodecyl sulfate or sodium dodecylbenzene sulfonate [55]. It turned out that the LDHs with low [M^2+^]/[M^3+^] ratios can achieve higher exfoliation yield. LDHs were rarely exfoliated in nonpolar solvents (e.g., hexane), while they were delaminated best in alcohols, such as 1-butanol, 1-octanol, and so on.

**Fig. 5:**
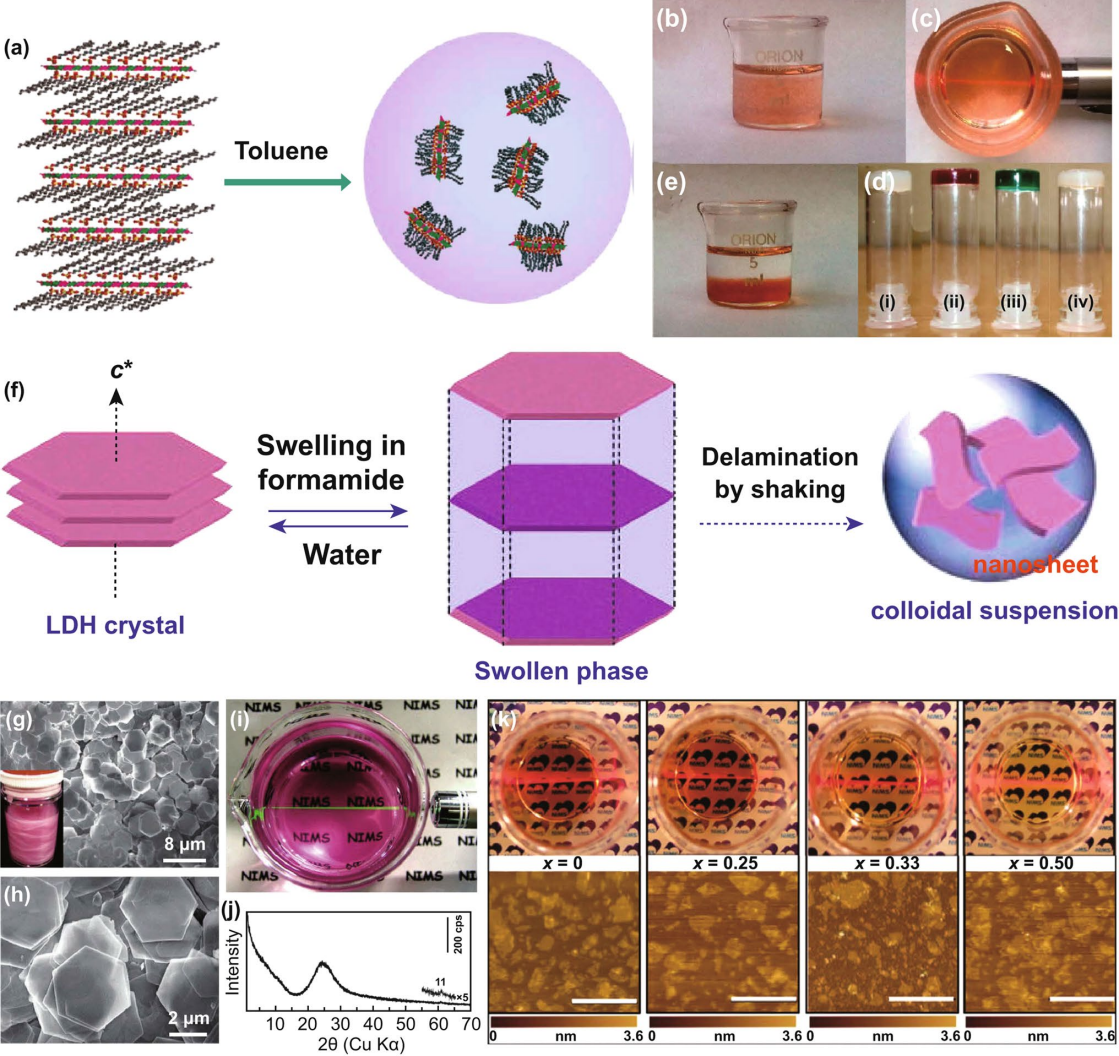
**a** Delamination process of a surfactant-intercalated LDH by toluene. **b**, **c** Photograph and Tyndall effect of Co–Al LDH dispersion exfoliated by toluene. **d** Test-tube inversion test demonstrating the formation of toluene gels for dispersion of Mg–Al LDH (i), Co–Al LDH (ii), Ni–Al LDH (iii), and Zn–Al LDH (iv). **e** The Co–Al LDH dispersion exfoliated by toluene after 7 days. Reproduced with permission from Ref. [56]. Copyright 2011 American Chemical Society. **f**–**j** Delamination process, low- and high-magnification SEM images, photograph and XRD pattern of the Co–Al–CO_3_ LDH sample and the suspension. Reproduced with permission from Ref. [53]. Copyright 2006 American Chemical Society. **k** Photographs of nanosheet suspensions of the Co_1−*x*_–Ni_*x*_ LDHs (*x *= 0, 0.25, 0.33 and 0.5) and the corresponding AFM images. Reproduced with permission from Ref. [57]. Copyright 2009 American Chemical Society

Toluene was also studied as the dispersant of DS^−^-intercalated LDH for liquid delamination [56]. As depicted in Fig. 5a–e, after being stirred in toluene and sonicating for 5 min, DS^−^-intercalated Mg_0.67_/Al_0.33_-LDH and Co_0.67_/Al_0.33_-LDH were rapidly exfoliated into monolayers with a clear transparent dispersion which showed a clear Tyndall light scattering effect. The exfoliation mechanism was proposed based on molecular dynamics simulation. For the LDH compounds incorporated with long-chain surfactant molecules, the Van der Waals interactions between chains anchored onto adjacent host layers play a role in holding the sheets together. The treatment with the solvents was expected to weaken or disrupt such van der Waals interactions and the DS^−^ converted the hydrophilic LDHs into hydrophobic, which promotes solvation with nonpolar solvent molecules, such as toluene.

Formamide is also a common solvent to perform liquid exfoliation. Hibino and Jones firstly reported the exfoliation of LDHs in formamide by creating a desirable interlayer environment to intercalate a large volume of solvent [58]. They prepared Mg_*n*_/Al_*k*_-LDHs intercalated with different amino acid anions, including glycine, seine, and l-aspartic acid. Diverse polar solvents were examined as dispersants, such as water, ethanol, acetone, formamide, ethylene glycol, and diethyl ether. Among all the combinations, they found that the glycine and formamide led to the optimum result. When 0.03 g Mg_3_/Al–glycine–LDH was mixed with 10 mL formamide under stirring, rapid exfoliation occurred in a few minutes. They also reported the modification of Mg–Al LDHs with various amino acids to create an interlayer environment that was suitable for solvation of formamide, which would break the hydrogen bonding network and lead to delamination [59].

Liu et al. performed a systematic research of the delamination of Co–Al LDHs in formamide (Fig. 5f–j) [53]. Hexagonal Co–Al–CO_3_ LDH platelets of 4 µm in lateral size were firstly prepared by the urea method and then converted to Cl^−^-LDH by treating with a NaCl–HCl mixed solution [60]. After that, Co–Al LDH intercalated with diverse anions was obtained via an anion exchange process employing corresponding salts, including NO_3_^−^, ClO_4_^−^, acetate, lactate, dodecyl sulfate, and oleate. The exchanged product (0.1 g) was mixed with 100 mL formamide and agitated vigorously in a mechanical shaker at a speed of 160 rpm for 2 days. A pink transparent suspension was attained, containing well-defined nanosheets with a lateral size up to 2 µm. The height profile of AFM revealed that the nanosheets possessed a fairly flat morphology with an average thickness of ~ 0.8 nm, which can be explained as the sum of crystallographic thickness of the LDH layer (0.48 nm) and an absorbed monolayer of formamide molecules (~ 0.3 nm) [61]. Such thickness value manifested the unilamellar structure of the exfoliated nanosheets. Liang et al. developed a topochemical synthesis of Co–Ni LDHs from brucite-like Co–Ni hydroxide with bromine as an oxidizing agent. Excess bromine in acetonitrile promoted partial oxidation of Co^2+^ into Co^3+^ [57]. Through the subsequent ethanol-assisted anion exchange process, a variety of inorganic and organic anionic forms of Co–Ni LDHs were achieved. The as-prepared NO_3_^−^-intercalated CoNi LDHs without substantial carbonate contamination were successfully delaminated into unilamellar nanosheets bearing positive charges upon contact with formamide (Fig. 5k). Different characteristic colors were presented for the translucent suspensions of nanosheets, depending on the variable Co/Ni ratios. It is worth noting that LDHs could also be delaminated in aqueous solution. Iyi and coworkers added aqueous zwitterion solution into ClO_4_^−^-intercalated MgAl-LDH and successfully attained semitransparent colloidal suspension [62].

##### Plasma Exfoliation

The concept of plasma was firstly proposed by Langmuir in 1928 [63], which has induced enormous research interest in materials synthesis and surface modifications during the past decades [64–68]. Besides being used to modifying surface [69], creating defects [70], and synthesizing materials [71], plasma has also been developed as technologies to exfoliate layered compounds, including graphite [72], black phosphorus [73], and also LDHs [74]. Wang et al. for the first time reported the efficient exfoliation of ultrathin CoFe LDH nanosheets by means of water plasma [75]. The plasma partially etched the interlayer anions and destroyed the electrostatic interactions between the host layers, thus resulting in fast exfoliation and simultaneously generating multivacancies in the as-exfoliated LDH nanosheets. Later on, they also successfully realized the exfoliation of bulk CoFe LDHs into ultrathin nanosheets by N_2_ plasma [76]. CoFe LDHs were firstly prepared by hydrothermal reaction and then treated by N_2_ plasma for 60 min. After the N_2_ plasma treatment, bulk CoFe LDHs were delaminated into ultrathin nanosheets. Numerous atomic-sized holes were also produced in the meantime with more exposed edge sites. As revealed by the height profiles of AFM, the thickness was decreased from ~ 20 to ~ 1.6 nm. Many holes were also observed in the basal plane of the exfoliated LDH nanosheets induced by the etching effect of plasma.

## Structure Modulation toward Application in Oxygen Evolution Reaction

Transition metal-based LDH materials have attracted considerable interest as the promising OER catalysts in alkaline system, due to their unique 2D layered structure, electronic property, and low cost. However, the catalytic performance of LDHs for OER is still restricted by their low electrical conductivity (10^−13^–10^−17^ S cm^−1^), limited active sites, inferior thermostability, and weak adsorption of oxygenated intermediates [77]. Therefore, the following parts will mainly discuss a series of effective strategies for obtaining remarkable electrocatalytic activities toward OER.

### Doping

LDHs can be regarded as metal-doped monometallic hydroxides [11]. Monometallic hydroxides (e.g., nickel hydroxides and cobalt hydroxides) were usually synthesized under ambient conditions in air, which would lead to the inevitable oxidation of Ni^2+^ or Co^2+^ into their trivalent state. Therefore, positively charged Ni^3+^-doped Ni(OH)_2_ and Co^3+^–Co(OH)_2_ would be produced under the regular conditions from the transformation of monometallic hydroxides [78–81]. Although many studies have been devoted to monometallic Ni(OH)_2_- and Co(OH)_2_-based materials for OER, their electrocatalytic activity is still not satisfactory. Introducing metallic dopants with various valent states into monometallic hydroxide is viewed as an ideal strategy to enhance their intrinsic catalytic activity because of the redistribution of localized *π*-symmetry electrons through bridging O^2−^ and endowing favorable adsorption/desorption of oxygen-containing species [82]. Besides, replacing some lattice oxygen in metal hydroxides with nonmetal elements (e.g., B, N, P, S, F) is also a promising strategy to modulate the electronic structure of hydroxides. Incorporating other metal or nonmetal elements would efficiently improve the conductivity and in turn promote the electrochemical performance of LDHs for water splitting. Hu et al. prepared NiCo and CoCo LDHs with Br^−^ anions via a topochemical approach. The electrochemical measurements showed that the NiCo LDH exhibited lower overpotential and Tafel slopes for OER than that of pure CoCo nanosheets, which can be attributed to the synergistic effects between different metal ions [12]. Lu et al. demonstrated a facile and practical pathway to fabricate 3D porous sulfur-incorporated NiCoFe LDH nanosheets on carbon cloth and achieved superb electrocatalytic activity and stability for OER [83]. The enhanced performance is attributed to the hierarchical nanostructure and sulfur doping which endows the electrode with high electrical conductivity [83].

Due to the versatile composition of LDHs, a large number of active sites can be easily incorporated into the structure of LDHs to modulate the electronic structure and active species. However, the OER activity of bimetallic LDHs is still not close to their optimal state, which can be further enhanced by doping the third metallic element with the formation of trimetallic LDHs. The additional doping can not only modify the electronic configuration of bimetallic LDHs, but also create synergetic effects between host layers and the dopants, which would enhance the intrinsic activity for OER and bring new properties toward other electrochemical applications, such as hydrogen evolution reaction (HER) and oxygen reduction reaction (ORR). Mukerjee et al. produced high-surface-area Ni–Fe–Co films on Raney nickel supports and found that such catalysts exhibited enhanced OER mass activity as compared to the Ni–Fe catalyst. The enhancements were ascribed to two aspects: (1) the charge transfer effect leads to the formation of conductive Ni^III^OOH species at lower overpotential, therefore activating the Fe sites that are inaccessible to electron transfer in the nonconductive Ni^II^(OH)_2_. (2) Introducing Co effectively induces the shrinkage of the Ni and Fe local geometry and likely results in an optimized Fe–OH/OOH bond strength as revealed by XAS analysis [84]. Duan et al. fabricated Mn^4+^-doped NiFe LDHs by a simple coprecipitation method at room temperature [85]. The as-prepared NiFeMn LDHs presented a flowerlike morphology assembled by the ternary LDH nanosheets with an average lateral size of 50 nm and thickness of 3.7 nm. Such Mn^4+^-doped NiFeMn LDH exhibited lower onset potential of 200 mV and faster OER current increase as compared to undoped NiMn LDH and NiFe LDH. A smaller Tafel slope of 47 mV dec^−1^ was also observed. A high stability was also achieved for delivering constant current density of 20 mA cm^−2^ over 15 h. The high catalytic activity is ascribed to the intrinsic electrocatalytic active Ni^2+^ and Fe^3+^ and the synergy between Mn dopants and these active sites. The DFT calculation proved that Mn^4+^ doping could narrow the bandgap of NiFe LDH with exposing more conductive electronic structure and thus improve the electric conductivity. The resistance of NiFeMn LDH disk-shaped pellet is 1.6 × 10^3^ Ω sq^−1^ that is lower than that of the corresponding NiFe LDH (2.2 × 10^3^ Ω sq^−1^). Moreover, doping Mn into NiFe LDH can also facilitate the formation of intermediates of ^*^O and ^*^OH, accelerating the OER catalytic process.

### Introduction of Cavity

**Fig. 6:**
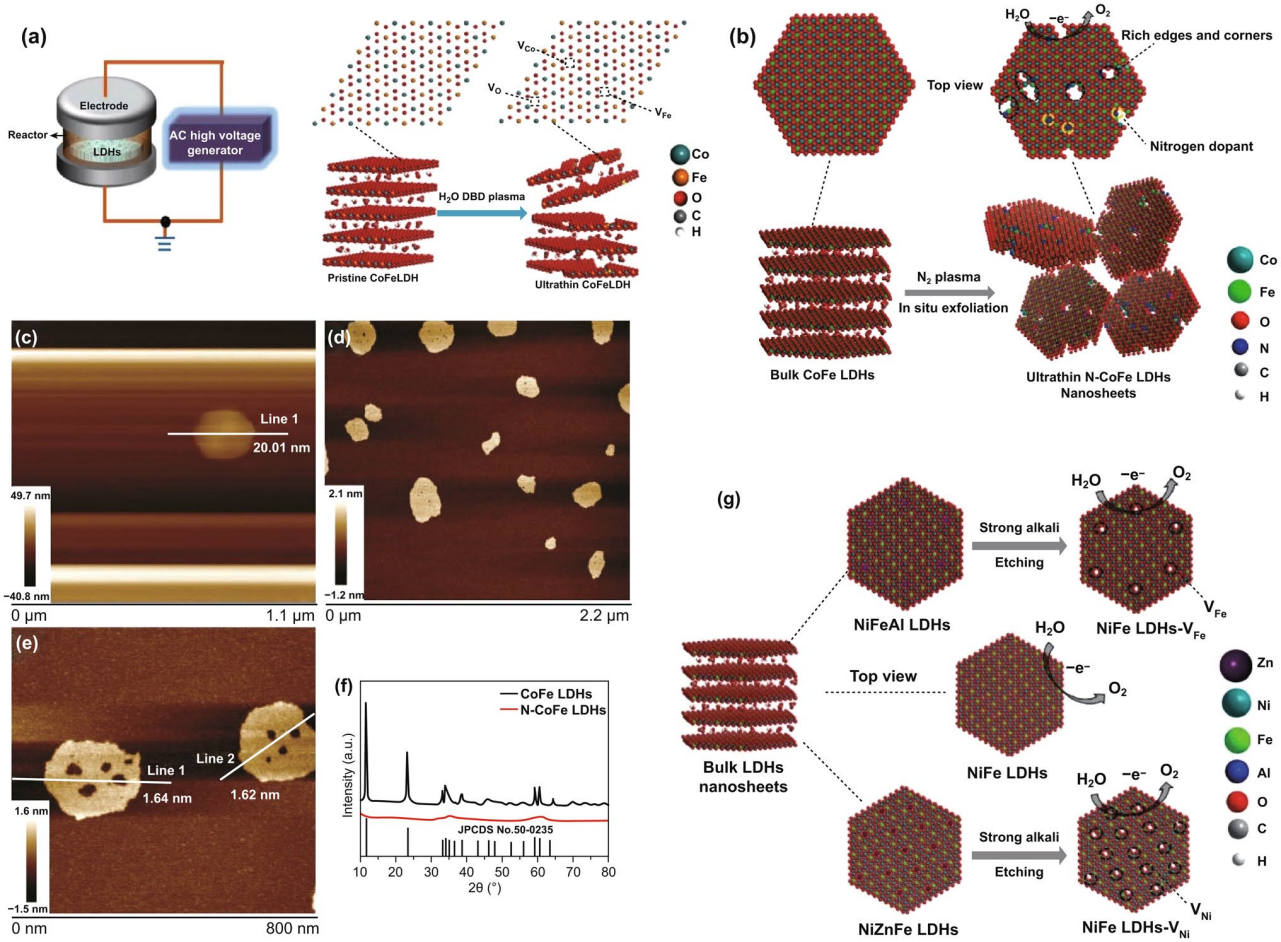
**a** Schematic illustration of the water-plasma-enabled exfoliation process of CoFe LDH nanosheets. Reproduced with permission of Ref. [75]. Copyright 2017 WILEY–VCH Verlag GmbH & Co. KGaA, Weinheim. **b** Illustration of the exfoliation process of bulk CoFe LDH into nanosheets by N_2_ plasma. **c** AFM image of the bulk CoFe LDH. **d**–**f** AFM images and XRD pattern of the ultrathin N-CoFe LDH nanosheets. Reproduced with permission of Ref. [76]. Copyright 2018 WILEY–VCH Verlag GmbH& Co. KGaA, Weinheim. **g** The synthesis of NiFe LDHs-V_Fe_ and NiFe LDH-V_Ni_ by strong alkali etching LDHs. Reproduced with permission of Ref. [89]. Copyright 2018 WILEY–VCH Verlag GmbH& Co. KGaA, Weinheim

Introducing cavities have been regarded as efficient strategies to tune the coordination valence and surface chemical environment of electrocatalysts [86–88]. Wang et al. applied water and N_2_ plasma to exfoliate bulk CoFe LDHs into ultrathin nanosheets [75, 76]. As illustrated in Fig. 6, cavities were introduced into the nanosheets during the exfoliated process. With the assistance of N_2_ plasma, nitrogen could also be doped to alter the surrounding electronic arrangement of the increased reactive sites facilitating the adsorption of OER intermediates [76]. In addition, introducing defects could further effectively tune the electrocatalytic activity of reactive sites by increasing the number of dangling bonds around reactive sites and decreasing the coordination number of reactive sites. Later on, they successfully realized the selective formation of cation vacancies in NiFe LDH nanosheets (Fig. 6g) [89]. The as-prepared NiFe LDHs-V_Fe_ and NiFe LDH-V_Ni_ electrocatalysts exhibited outstanding activity for OER, which exhibits a superior stability with almost no decay of the LSV curves after 2000 cycles [76]. The high performance may be ascribed to the introduction of rich iron and nickel vacancies in the LDH nanosheets and increase in the adsorbing capacity of OER intermediates by surface electronic tuning. DFT computational results further verified the OER performance can be enhanced by the Fe or Ni vacancies.

### Functional Nanoparticles-Decorated LDHs

**Fig. 7:**
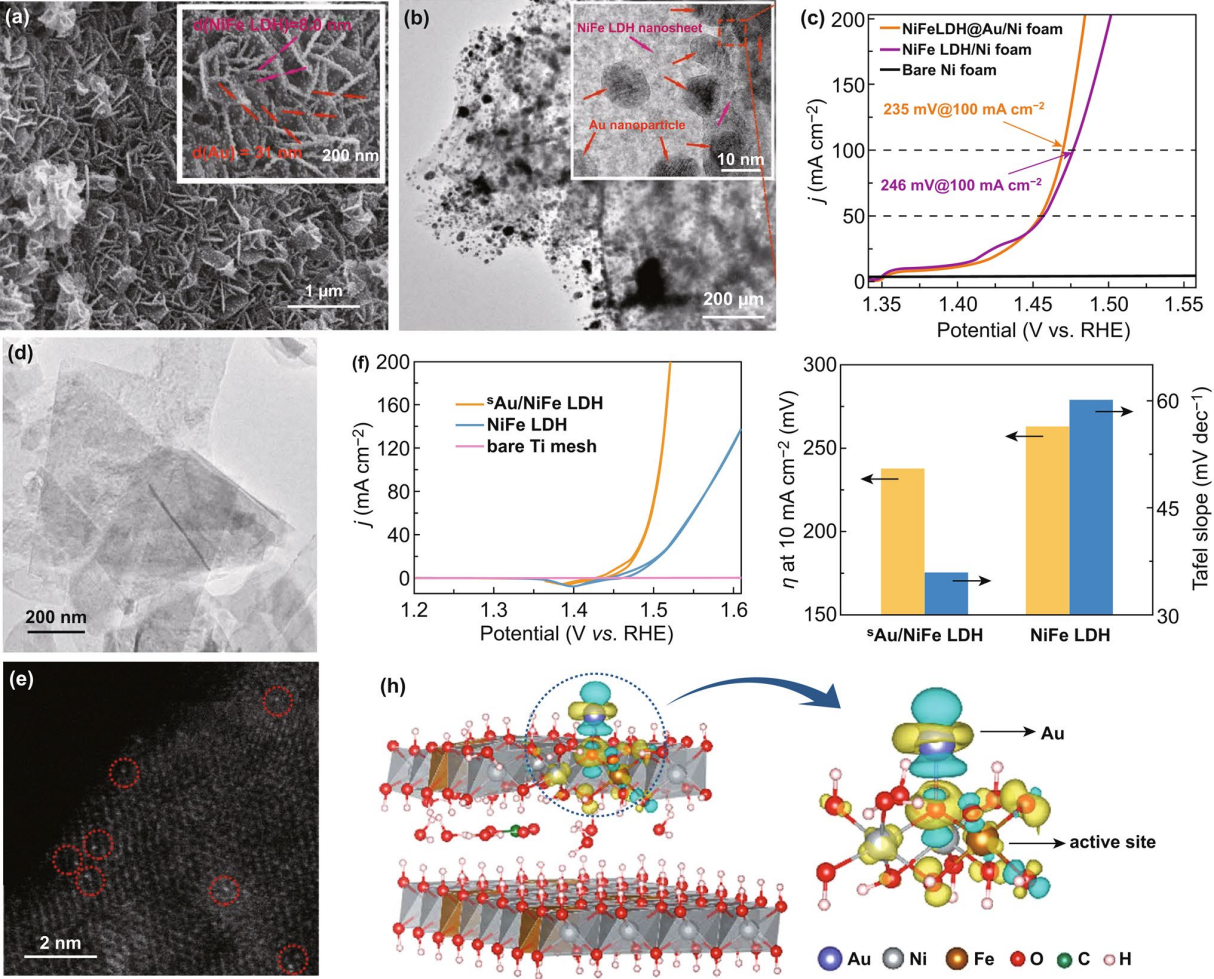
**a** SEM images, **b** TEM images of NiFe LDH@Au/Ni foam. **c** LSV curves of as-prepared catalysts at 1 mV s^−1^ in 1.0 M KOH. Reproduced with permission of Ref. [92]. Copyright 2017 American Chemical Society. **d** TEM image, **e** HAADF-STEM image of sAu/NiFe LDH. **f** Cyclic voltammetry (CV) curves of the catalysts at 5 mV s^−1^ with 95% iR compensation in 1 M KOH. **g** Overpotential (η) at 10 mA cm^−2^ and Tafel slope of pure NiFe LDH and sAu/NiFe LDH. **h** Differential charge densities of NiFe LDH supported with and without Au atom. Reproduced with permission of Ref. [97]. Copyright 2018 American Chemical Society

Recent works have reported that the LDHs decorated with functional nanoparticles can effectively enhance the electrocatalytic activity for OER [90–93]. Metal nanoparticles with high electrical conductivity can accelerate the electron transfer and increase the heterogeneous interaction of LDHs during the OER process [94]. Gao et al. fabricated Ni nanoparticles (Ni NPs) on the NiFe LDH ultrathin nanosheets (Ni NP/NiFe LDH) by a one-step hydrothermal method [93]. Within the Ni NP/NiFe LDH, the charge transfer resistance is reduced by highly conductive Ni NPs. Furthermore, the LDH nanosheets with highly exposed surface provide abundant catalytically active sites, and the intimate contact between Ni NPs and NiFe LDH forms the profitably synergic effects. Such Ni NP/NiFe LDH catalyst reveals high catalytic performance for OER due to aforementioned superiorities. Similarly, Zhu et al. reported Au-supported NiFe LDHs arrays on Ni foam (NiFe LDH@Au/Ni foam) prepared by hydrothermal reaction combined with chemical deposition (Fig. 7a, b) [92]. As an OER catalyst, NiFe LDH@Au/Ni foam requires very low overpotentials of 221, 235, and 270 mV to reach the current densities of 50, 100, and 500 mA cm^−2^ in alkaline solution, respectively, showing a remarkable OER performance (Fig. 7c). Besides the high conductivity of Au, the high catalytic activity for OER can be attributed to the following reasons. On the one hand, Au with high electronegativity can adsorb more electrons to generate and stabilize Ni^3+^ with high oxidation state, thus improving the OER efficiency. On the other hand, strongly electrophilic Ni^3+^ facilitates the formation of the hydroperoxy species (OOH) as the key intermediates for O_2_ evolution. Recent studies have focused on the single-atom metal-modified electrocatalysts which show higher electrocatalytic activity compared with nonmodified ones [66, 95, 96]. Zhang et al. developed a single-atom Au (0.4 wt%)-decorated NiFe LDH (sAu/NiFe LDH) electrocatalyst with a sixfold enhancement on the OER activity (Fig. 7d, e) [97]. After dispersing atomic Au on NiFe LDH, the overpotential of Au/NiFe LDH was reduced to 0.21 V, close to the calculated result (0.18 V) (Fig. 7f, g). The excellent OER activity is ascribed to the charge redistribution of active Fe and its surrounding atoms, which is induced by the neighboring Au atom on the NiFe oxyhydroxide generated from LDH and stabilized by CO_3_^2−^ and H_2_O during the OER process. The calculation results indicate that a net Au-to-LDH charge redistribution generated by the integrated charge density difference can transfer to surrounding atoms (e.g., O, Ni, and Fe), promoting the adsorption of OH^−^ and optimizing the adsorption of intermediates (e.g., O* and OOH*), thus reducing the overpotential from O* to OOH* in the rate-limiting step (Fig. 7h). As mentioned above, the formation of heterostructures between LDHs and metal nanoparticles is considered beneficial to enhance OER activity. Francisco and coworkers integrated Rh species with NiFe LDH, whereas Rh acts as oxidized dopants and metallic clusters (< 1 nm), which can dramatically improve OER kinetics with overpotential 7 and 35 mV smaller than that of NiFe LDH at 10 and 100 mA cm^‒2^, respectively [98]. Song et al. reported a facile one-step method synthesizing Ru atoms supported on a monolayer NiFe LDH with precise location instead of random dispersion [99]. The overpotential of such Ru/mono-NiFe is 104 and 134 mV lower than that of mono-NiFe and bulk-NiFe, respectively. Such catalysts also showed high stability with no obvious decline after 600 cycles.

**Fig. 8:**
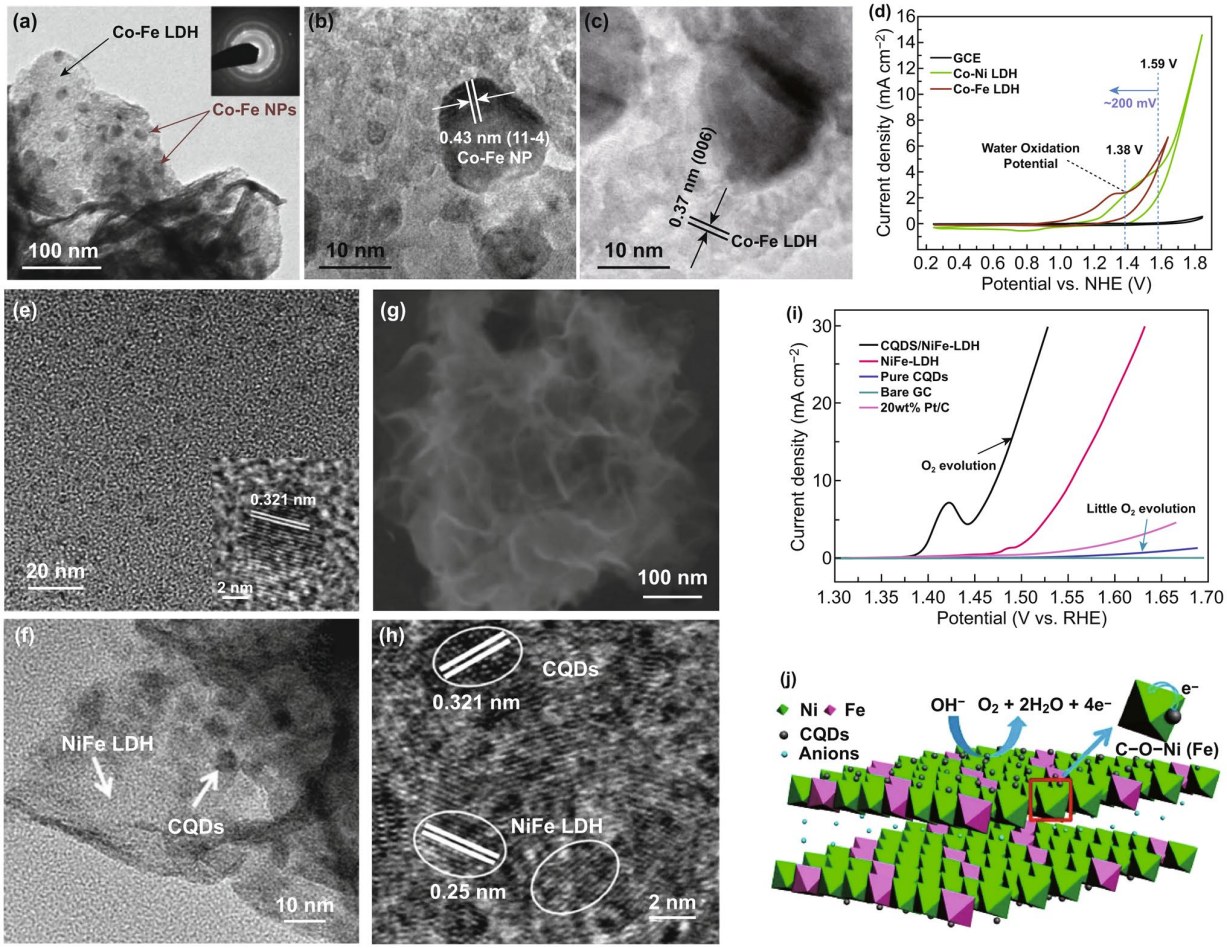
**a** TEM and SAED images (inset of **a**), **b** and **c** HRTEM images of Co–Fe LDH decorated with Co–Fe hydroxide NPs. **d** CV curves of the three catalysts at 20 mV s^−1^ in 0.1 M phosphate buffer. Reproduced with permission of Ref. [100]. Copyright 2015 The Electrochemical Society. **e** TEM image and HRTEM image (inset of **e**) of CQD. **f** TEM image, **g** SEM image, **h** HRTEM image of the CQD/NiFe LDH hybrid. **i** LSV curves of as-prepared catalysts at 5 mV s^−1^ in 0.1 M KOH. **j** Schematic model of the roles of CQDs in the CQD/NiFe LDH hybrid catalyst. Reproduced with permission of Ref. [101]. Copyright 2014 American Chemical Society

In addition to the metal nanoparticles, the incorporation of nonmetal nanoparticles to LDHs has also been considered as an effective approach to improve the electrocatalytic performance [100, 101]. Valdez et al. found that the OER performance of CoFe LDHs can be enhanced by modifying with CoFe hydroxide nanoparticles, due to the synergistic effect between the CoFe LDHs and CoFe hydroxide nanoparticles (Fig. 8a–d) [100]. The Fe with high dispersibility could change the electronic properties of Co–Fe LDH catalysts. The interaction between the electrons of CoFe LDH surface and those from Fe or Co of the nanoparticles lowers the OER overpotential. Carbon quantum dot (CQD) with a small particle size (< 5 nm) is a new class of nanocarbon, which shows high conductivity, fast electron transfer, and electron reservoir properties [102–104]. Kang et al. combined NiFe LDHs with CQDs to construct the CQD/NiFe LDH hybrids by a two-step solvothermal reaction, in which the CQDs (~ 5 nm) are anchored on the ultrathin NiFe LDH nanoplates with the thickness of around 1 nm (Fig. 8e–h) [101]. The CQD/NiFe LDH hybrids achieve a small overpotential of 235 mV at the current density of 10 mA cm^−2^ in 1 M KOH, showing the high OER activity (Fig. 8i). The strong bonding and interaction between NiFe LDH nanoplates and CQDs accelerate the charge transport between them, leading to the enhanced catalytic activity for OER (Fig. 8j).

### LDHs/Conductive Components Hybrids

**Fig. 9:**
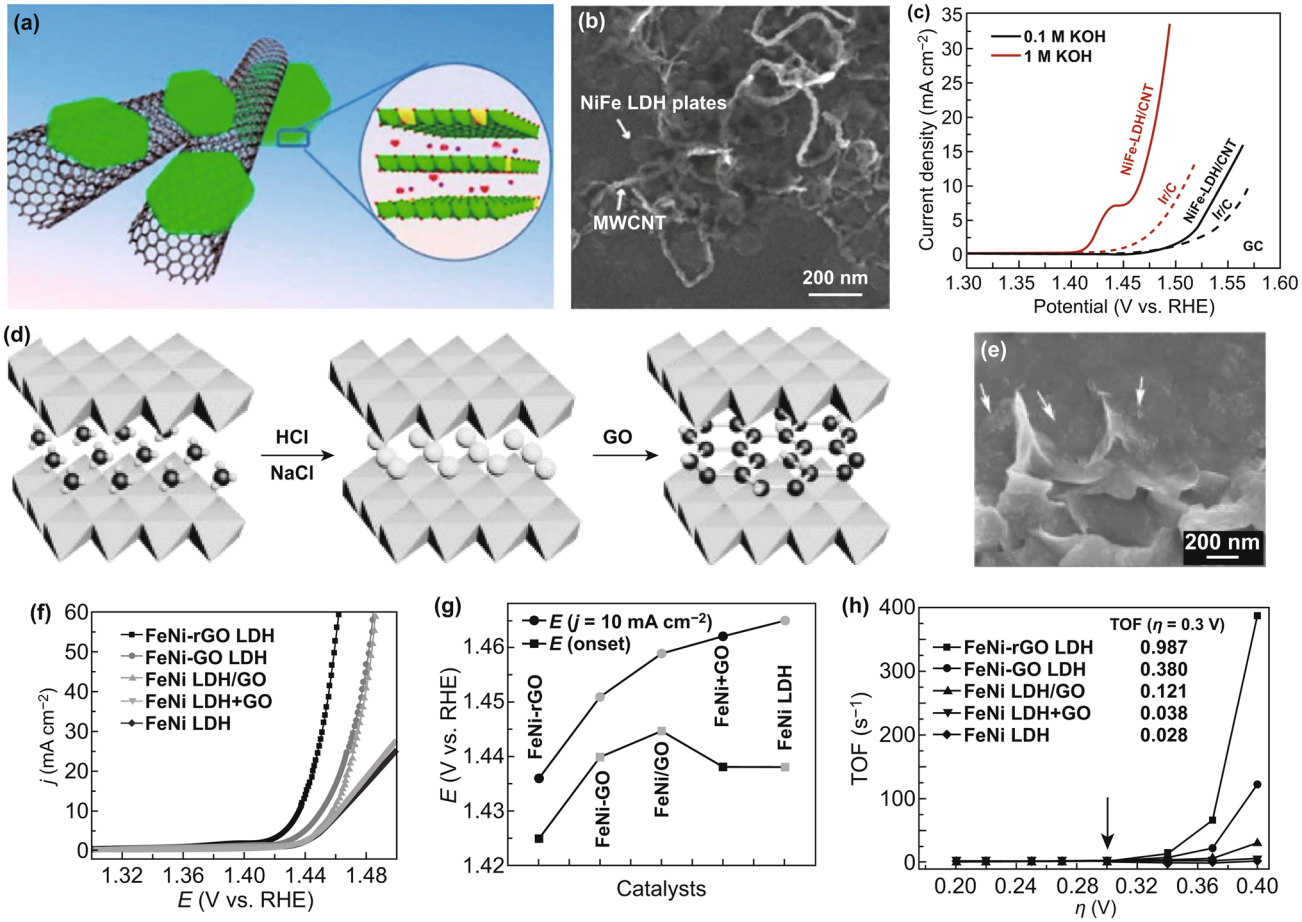
**a** Schematic diagrams of the NiFe LDH/CNT hybrid architecture and LDH crystal structure. **b** SEM image of NiFe LDH/CNT hybrid catalyst. **c** 95% *iR*-corrected LSV curves of the three catalysts at 5 mV s^−1^ in 0.1 and 1 M KOH. Reproduced with permission of Ref. [108]. Copyright 2013 American Chemical Society. **d** Fabrication process, **e** SEM image of FeNi-GO LDHs. **f** LSV curves of the catalysts at 5 mV s^−1^ in 1 M KOH. **g** Onset potentials and potentials at 10 mA cm^−2^ of the as-prepared catalysts during the OER process. **h** TOF values of the catalysts as a function of overpotential and TOF values at an overpotential of 0.3 V (inset of **h**). Reproduced with permission of Ref. [111]. Copyright 2014 WILEY–VCH Verlag GmbH& Co. KGaA, Weinheim

**Fig. 10:**
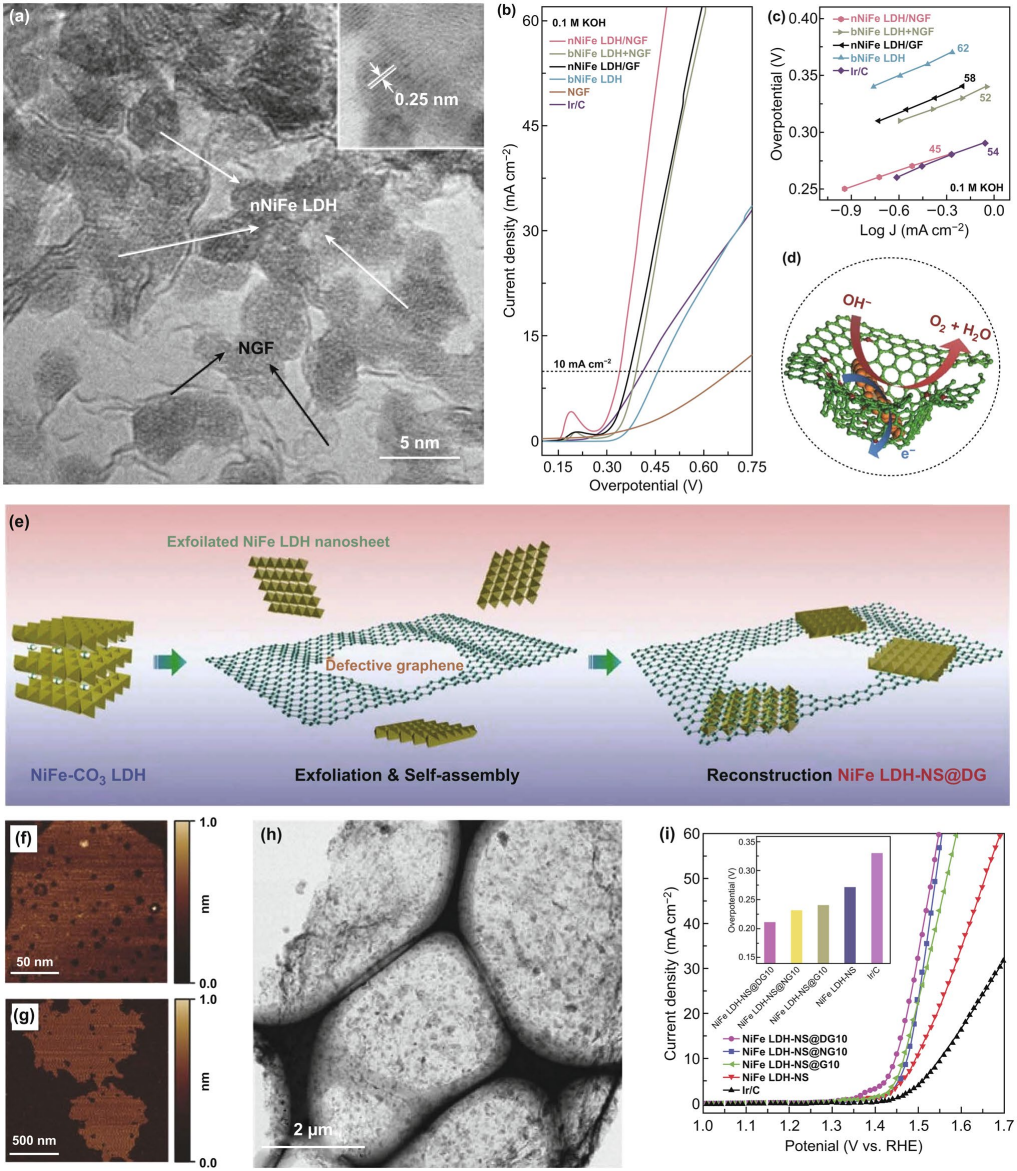
**a** TEM image and HRTEM image (inset of **a**) of nNiFe LDH/NGF catalyst. **b** 95% *iR*-corrected LSV curves of the catalysts at 5 mV s^−1^ in 0.1 M KOH. **c** Tafel plots of the different catalysts for comparison. **d** Schematic diagram of the spatially confined nNiFe LDH/NGF hybrids. Reproduced with permission of Ref. [115]. Copyright 2015 WILEY–VCH Verlag GmbH& Co. KGaA, Weinheim. **e** Schematic diagram of the fabrication of NiFe LDH-NS@DG hybrid. AFM images of **f** exfoliated NiFe LDH-NS and **g** DG. **h** TEM image of NiFe LDH-NS@DG catalyst. **i** *iR*-corrected LSV curves of as-prepared catalysts at 5 mV s^−1^ in 1 M KOH and their overpotential at 10 mA cm^−2^ (inset of **i**). Reproduced with permission of Ref. [112]. Copyright 2017 WILEY–VCH Verlag GmbH& Co. KGaA, Weinheim

Despite the considerable progress in controlling the structure of LDHs-based OER catalysts, their development is still seriously hindered by the poor conductivity and confined space for catalytic reactions. To further boost the catalytic performance of LDHs toward OER, it is necessary to combine LDHs with new components. Carbon-based materials (*e.g.,* carbon nanotubes (CNTs) and graphene) as catalyst supports have been widely used in various catalytic systems, because of the beneficial physical and chemical properties including high electronic conductivity, good mechanical strength, outstanding thermal stability, and large specific area [14, 105]. Constructing LDH-based catalysts on carbon-based materials can significantly improve the electrocatalytic performance for OER. As one-dimensional conductors, CNTs possess good electrocatalytic activities, which can be used to further boost OER activities of LDHs by functionalization [101, 106, 107]. Dai et al. successfully grew ultrathin NiFe LDH nanoplates on mildly oxidized multiwalled CNTs by solvothermal treatment (Fig. 9a, b) [108]. The electronic conductivity and dispersion of NiFe LDHs are improved by introducing multiwalled CNT support. Moreover, the exposure of active sites can also be increased. The strong interaction between NiFe LDHs and CNTs ensures the fast electron transfer during the OER process. As a result, the NiFe LDH/CNT catalyst with a high TOF value shows high catalytic activity and durability for OER in basic solution (Fig. 9c). Among various carbon-based materials, graphene nanosheet has been considered an ideal 2D carbon material [109, 110]. After functionalization of surface, the obtained graphene oxide (GO) has rich oxygenic groups which favor the assembly between GO nanosheets and LDH layers through electrostatic attraction force, thus resulting in the formation of the interlayered hybrid sheets [111]. These hybrid sheets exhibit an enhanced electrocatalytic activity, owing to the conductive graphene sheets, highly exposed and dispersed active sites, and strong synergistic effects. Up to now, graphene and its derivatives (e.g., GO, reduced graphene oxide (rGO), and heteroatom-doped graphene) have been extensively utilized to couple with LDHs [19, 111–113]. Yang et al. fabricated FeNi-LDH/GO hybrid nanosheets (FeNi-GO LDH) via the alternate stack of the GO layers and FeNi double hydroxide cation layers (Fig. 9d, e) [111]. The FeNi-GO LDH catalyst shows high OER activity with low overpotential (0.21 V) and small Tafel slope (~ 40 mV dec^−1^). After reducing GO to rGO, the overpotential for OER of as-prepared FeNi-rGO LDH catalyst can be further decreased to 0.195 V, while its TOF value at the overpotential of 0.3 V is as high as 0.98 s^−1^ (Fig. 9f–h). The excellent electrocatalytic performance for OER of the NiFe-rGO LDH is mainly attributed to the FeNi double hydroxide with high OER catalytic activity and the rGO layers with high conductivity. Based on the electronegativity theory, the introduction of heteroatom-doped graphene favors the dispersibility and stability of 2D nanolayers on graphene [113, 114]. Wei et al. reported nanometer-sized NiFe LDHs grown on N-doped graphene framework (nNiFe LDH/NGF) catalysts (Fig. 10a) [115]. The defects and nitrogen dopant of graphene are beneficial to adsorb and anchor metal cations, and then the nNiFe LDHs nucleate and grow within the mesopores of graphene, finally obtaining the uniformly dispersed nNiFe LDHs in the N-doped graphene frameworks. The nNiFe LDH/NGF catalyst shows high catalytic activity and low Tafel slope for OER, profiting from fully exposed active sites, suppressed particle aggregation, intimate interfacial coupling, interconnected conductive network, and hierarchical porous structure (Fig. 10b–d). In recent years, the introduction of topological defects in carbon-based materials has proven to be an effective method for boosting the electrocatalytic performance [116]. For instance, Yao et al. coupled exfoliated Ni–Fe LDH nanosheet (NS) with defective graphene (DG) by electrostatic stacking (Fig. 10e–h) [112]. This hybrid catalyst needs a low overpotential (0.21 V) to achieve a current density of 10 mA cm^−2^ in the OER process, exhibiting the high electrocatalytic activity (Fig. 10i). The defects in graphene can not only directly serve as active sites but also offer more anchor sites to capture Ni and Fe atoms through the π–π interaction, thus leading to fast electron transfer kinetics, high catalytic activity, and stability (Table 1).

**Table 1:** Comparison of OER activities of different catalysts

Catalysts	Electrolyte	η@10 mA/cm^2^ (mV)	Tafel (mV dec^−1^)	Refs.
NiFe LDH	1 M KOH	347	67	[12]
NiFe LDH (exfoliated)	1 M KOH	302	40	[12]
Ni_2_CoFe LDH + GO	0.1 M KOH	345	74.5	[117]
Ni_2_Co^III^Fe-LDH/N-GO	0.1 M KOH	320	56.8	[117]
Ni_2/3_Fe_1/3_ LDH	1 M KOH	310	76	[118]
Ni_2/3_Fe_1/3_-GO	1 M KOH	230	42	[118]
NiCo LDH/CP (exfoliated)	1 M KOH	300	40	[119]
NiFe LDH (CO_3_^2−^)	1 M KOH	330	44.3	[120]
α-CoFe LDH	1 M KOH	295	52	[11]
NiFe LDH/N-doped graphene	0.1 M KOH	337	45	[115]
NiFe LDH nanosheets/3D carbon network	0.1 M KOH	380	77.9	[121]
NiFe LDH/graphitic mesoporous carbon	1 M KOH	320	57	[122]
3DGN/CoAl-NSs	1 M KOH	252	36	[123]
CoNi-LDH@PCPs	1 M KOH	350	58	[124]
FeNi-LDH/CoP/CC	1 M KOH	231 mV@20 mA/cm^2^	33.5	[125]

**Fig. 11:**
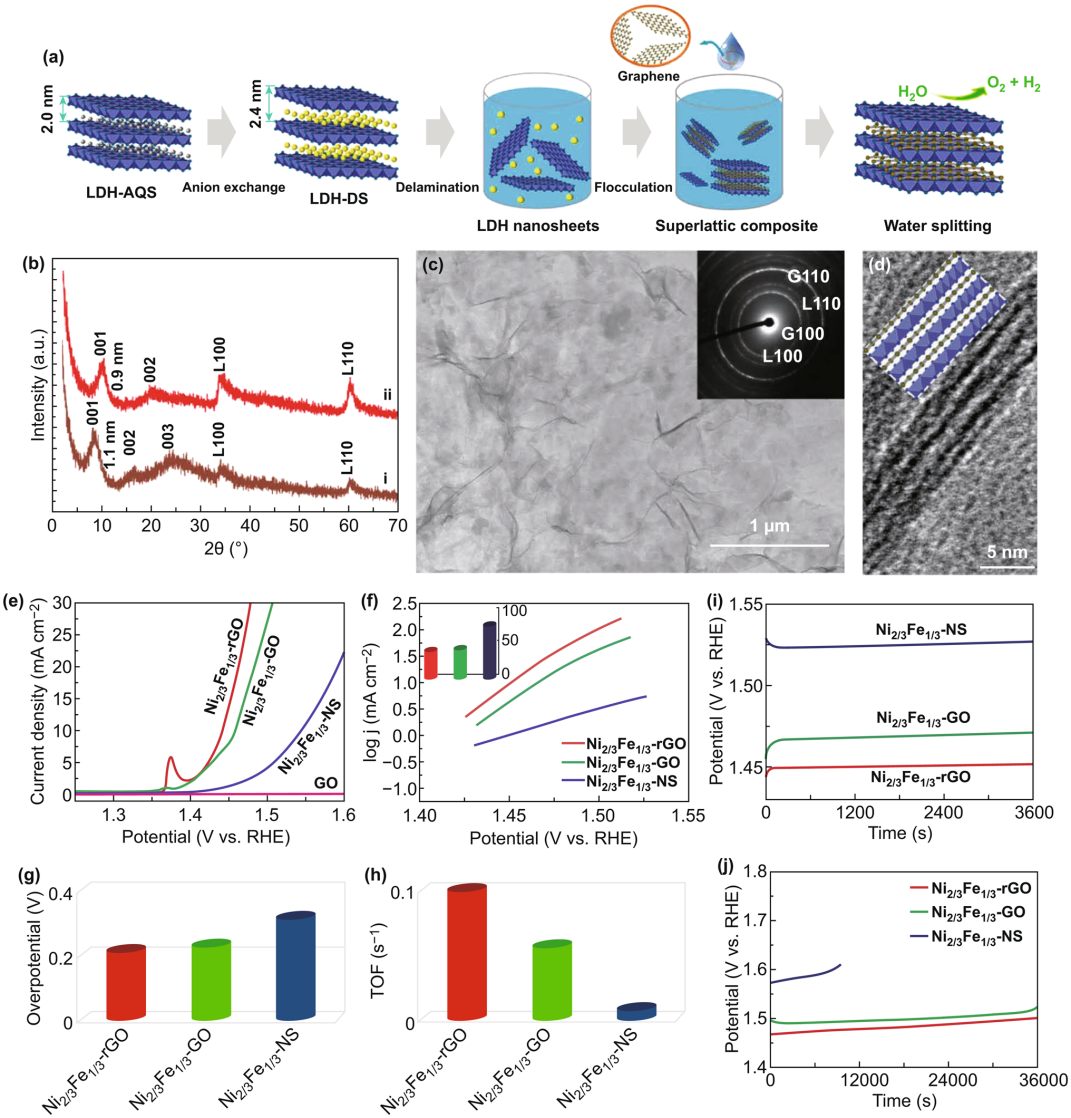
**a** Process of hetero-assembling Ni–Fe LDH nanosheets and graphene for water splitting. **b** XRD patterns of Ni_2/3_Fe_1/3_-NS/GO superlattice (i) and rGO (ii). **c, d** TEM and HRTEM images of the superlattice. **e–j** Electrochemical evaluation of the NiFe LDH/GO and NiFe LDH/rGO superlattices. Reproduced with permission of Ref. [118]. Copyright 2015 American Chemical Society

**Fig. 12:**
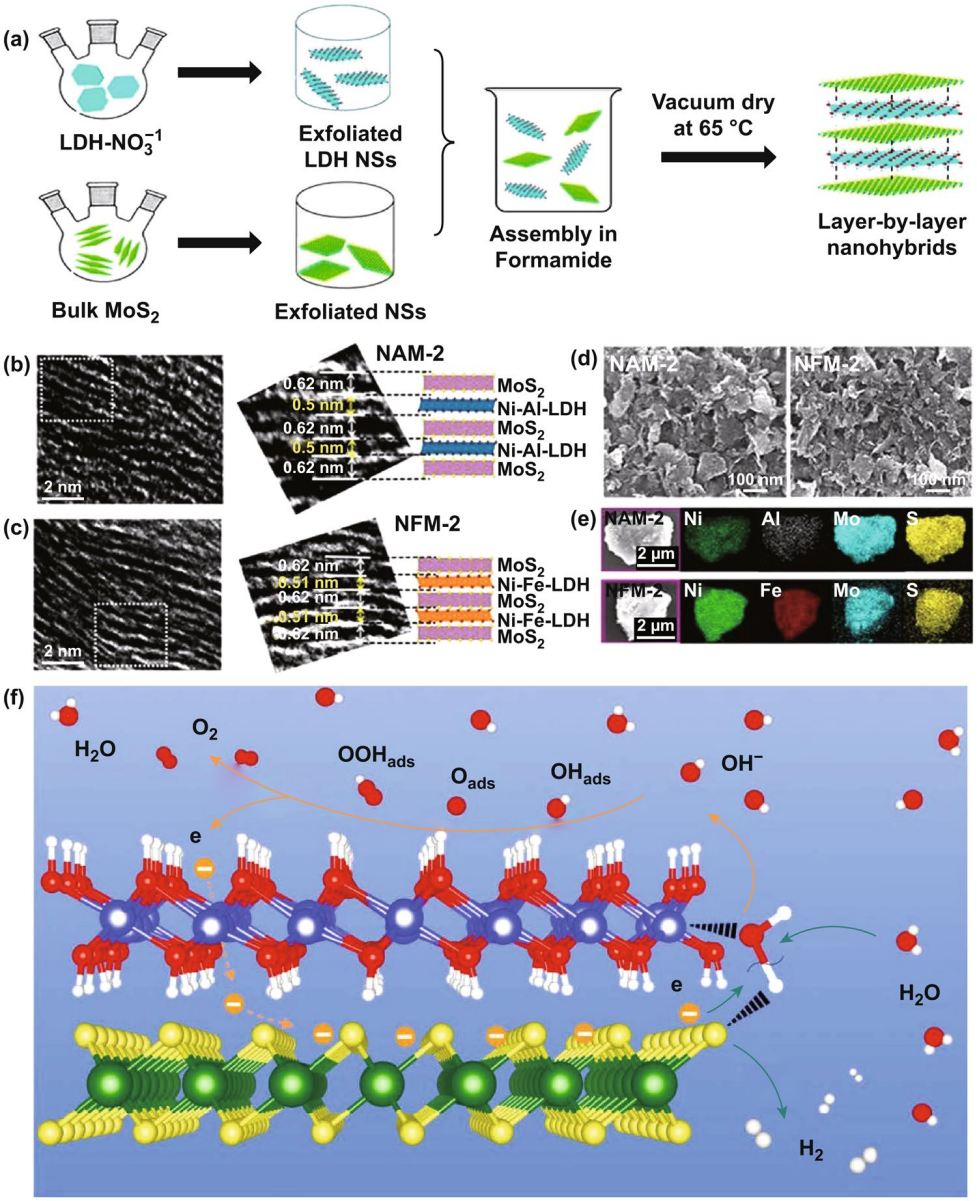
**a** Synthesis process, **b, c** cross-sectional TEM images, **d** FE-SEM images, and **e** EDS elemental mappings of NiAl LDH/MoS_2_ and NiFe LDH/MoS_2_. Reproduced with permission from Ref. [128]. Copyright 2018 American Chemical Society. **f** Schematic illustration of the electrocatalytic mechanism of water splitting on the interface of MoS_2_/LDH superlattice. Reproduced with permission from Ref. [129]. Copyright 2019 American Chemical Society

Moreover, Ma et al. sandwiched transition metal (Co–Al, Co–Ni) LDH nanosheets and graphene, for the first time, to form true superlattice lamellar nanocomposite by direct heterostacking [126]. Synergistic effect could be harvested based on the shortest distance and highest efficiency in charge transfer and ion diffusion during a redox process. The heteroassembly of superlattice structure would redistribute the electric charge between adjacent crystals in the stack. Moreover, the neighboring unilamellar nanosheets may induce lattice strain or structure change owing to the electrostatic interaction, thus resulting in synergetic effects to enhance the electrocatalytic activity. In addition, the conductivity of LDHs could be substantially improved by hybridization with conductive components in forming superlattice structure. To address the aggregate, insulating nature and poor stability issues of LDH in OER, Ma et al, proposed an approach of heteroassembly of hydroxide nanosheets and graphene to achieve full potential of the two complementing 2D counterparts [118, 127]. NiFe LDH was firstly synthesized by a homogeneous precipitation method in the presence of HMT and AQS. After being exfoliated into monolayers, they were assembled with GO and rGO into superlattice structure under stirring (Fig. 11a). In such heterostructure, graphene served as conducting paths to enhance charge transfer and mass transport due to its extremely high specific surface area (2600 m^2^ g^−1^ in theory) and high electrical conductivity (~ 10^6^ S cm^−1^). Figure 11b, c reveals the HRTEM images of the superlattice structures. The electrostatic face-to-face stacking of negatively charged graphene and positively charge Ni–Fe LDH nanosheets in alternating sequence at a molecular scale contributed to the direct interfacial contact between carbon and 3*d* transition metals, which significantly shortens the diffusion distance. Therefore, such superlattice achieved an extremely small overpotential of 210 mV at 10 mA cm^−1^ and Tafel plot of 40 mV decade^−1^. The high performance kept stable for 10 h without apparent degradation. Islam et al. synthesized bifunctional 2D superlattice electrocatalysts of alternating LDH transition metal dichalcogenide (TMD) heterostructures through interstratification with the exfoliated nanosheets (Fig. 12) [128]. The electrostatic self-assembly of 2D building blocks with opposite charge produces superlattice composites, such as Ni–Al LDH-MoS_2_ and Ni–Fe LDH-MoS_2_ superlattices. Density functional theory calculations predicted that the interfacial charge transfer between LDH and TMD components would be enhanced and thus improved the electrocatalytic activity. Xiong et al. fabricated MoS_2_/NiFe LDH superlattice by alternate restacking of MoS_2_ and NiFe LDH nanosheets which exhibited a low overpotential of 210 mV at 10 mA cm^−2^ for OER [129]. They assumed that the high performance can be attributed to the optimal adsorption energies of OER intermediates on the superlattice originated from a strong electronic coupling effect at the heterointerfaces (Fig. 12f).

**Fig. 13:**
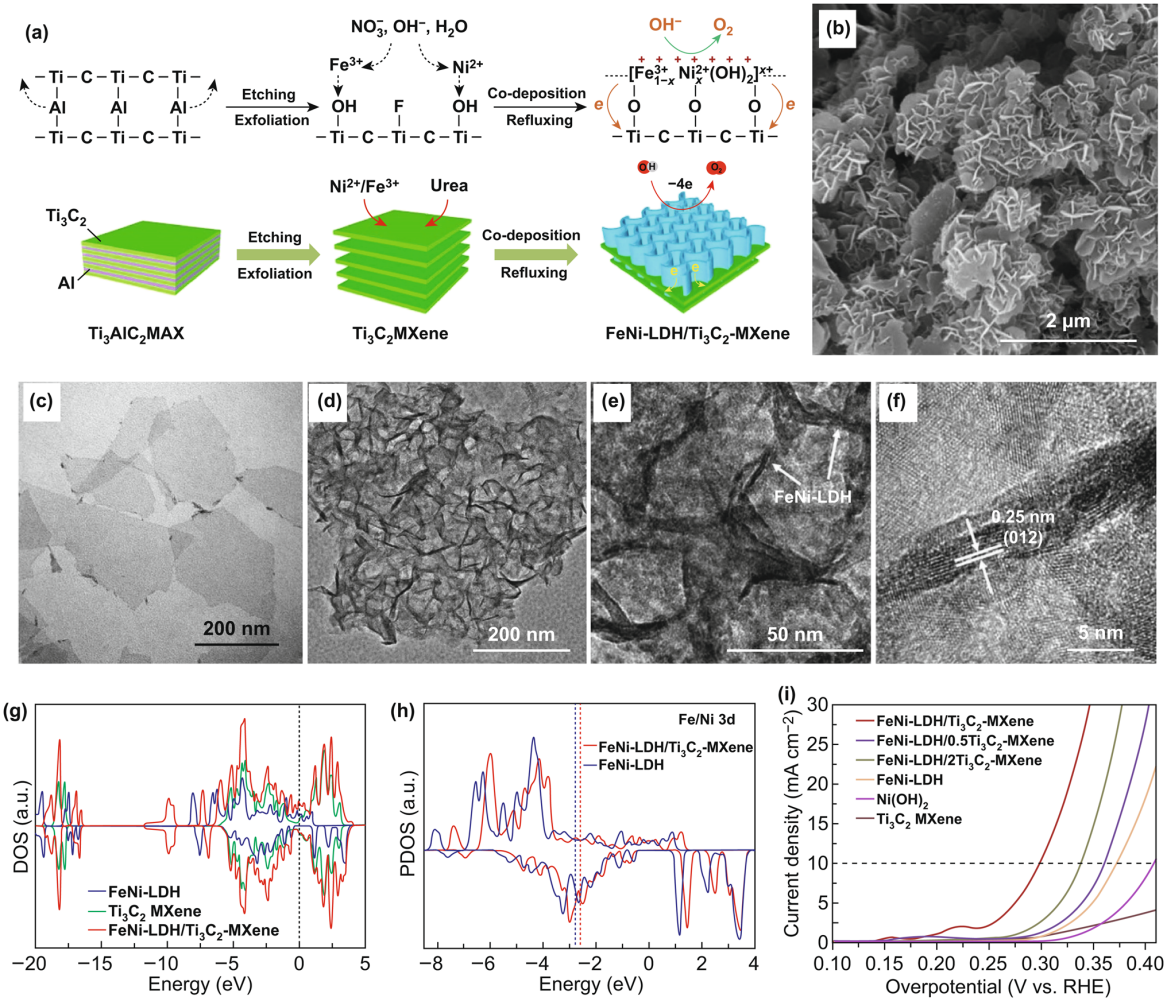
**a** Schematic diagram of the preparation of FeNi-LDH/Ti_3_C_2_ MXene hybrids. **b** SEM image, **c** TEM image, **d**–**f** HRTEM images of FeNi-LDH/Ti_3_C_2_ MXene. **g** density of states (DOS), **h** projected DOS (PDOS) of different samples. **i** LSV curves of as-prepared catalysts at 10 mV s^−1^ in 1 M KOH. Reproduced with permission of Ref. [130]. Copyright 2017 Elsevier Ltd

Besides aforementioned carbon-based materials, 2D layered transition metal carbides/carbonitrides (MXene) with metallic conductivity and hydrophilic surface became the promising candidates to assemble with LDHs for improving the catalytic performance [130]. Yu et al. developed a new-type hierarchical FeNi-LDH/Ti_3_C_2_ MXene hybrid electrocatalyst for OER, in which the interconnected porous network of FeNi-LDH nanoplates is in situ assembled on the ultrathin Ti_3_C_2_ MXene sheets (Fig. 13a) [130]. Figure 13b–f demonstrates that the FeNi-LDH nanoplates are firmly immobilized on Ti_3_C_2_ sheets to form a highly active network, preventing their adverse detachment/aggregation. In addition, the hydrophilic Ti_3_C_2_ MXene with high conductivity can not only effectively accelerate the ion transport and charge transfer, but also promote the Ni^2+^/Ni^3+, 4+^ redox process for OER. Within the LDH/MXene nanohybrids, the electron extraction from FeNi-LDH could improve the binding strength of O through shifting the d-band center of Ni/Fe atoms to higher energy, indicating the less occupied antibonding states between adsorbed O intermediates and FeNi-LDH (Fig. 13g, h). Owing to the above advantages, the FeNi-LDH/Ti_3_C_2_ MXene hybrid electrocatalyst shows fast reaction kinetics with high catalytic activity and good durability for OER (Fig. 13i).

### Construction of 3D Freestanding Electrodes Based on LDHs

**Fig. 14:**
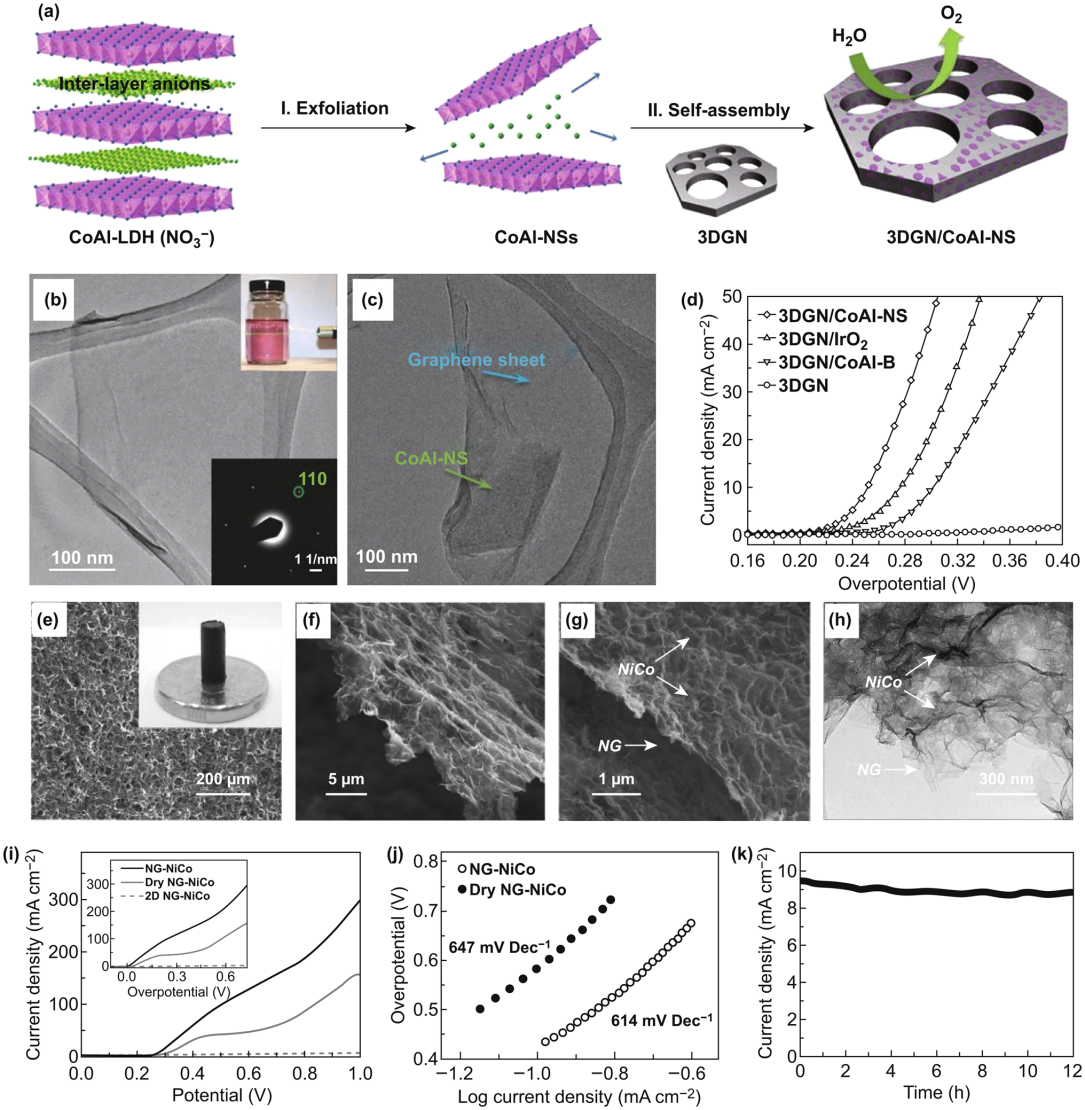
**a** Schematic diagram of the fabrication of 3DGN/CoAl-NS. **b** TEM image and SAED pattern (inset of **b** bottom) of the exfoliated CoAl-NS, the inset (top) of **b** is photograph showing Tyndall effect of CoAl-NSs solution under irradiation. **c** TEM image of 3DGN/CoAl-NS. **d** LSV curves of the catalysts at 1 mV s^−1^ in 1 M KOH. Reproduced with permission of Ref. [123]. Copyright 2016 WILEY–VCH Verlag GmbH& Co. KGaA, Weinheim. **e**–**g** SEM image and optical image (inset of **e**), **h** TEM image of NG-NiCo. **i** LSV curves of three catalysts at 50 mV s^−1^ in 0.1 M KOH. **j** Tafel plots for as-prepared samples. **k** *i*–*t* curve of NG-NiCo at 0.5 V versus Ag/AgCl. Reproduced with permission of Ref. [113]. Copyright 2013 WILEY–VCH Verlag GmbH& Co. KGaA, Weinheim

Constructing 3D architectures of LDH-based materials is very important for electrocatalytic application, due to their unique advantages including more active sites for electrocatalysis, facilitated penetration of electrolytes, and fast transport of electrons/ions [11, 131, 132]. Therefore, the fabrication of LDHs on 3D conductive supports to form 3D hierarchical architectures can substantially enhance the catalytic performance due to their synergistic properties. Zhang et al. combined single-layer CoAl LDH nanosheets with 3D graphene network to fabricate a 3DGN/CoAl-NSs catalyst for OER via the self-assembly method (Fig. 14a–c) [123]. The catalytic activity and durability of the as-prepared 3D GN/CoAl-NS catalyst are comparable or even better than those of many LDH-based OER catalysts. The 3DGN/CoAl-NS reveals a low Tafel slope of 36 mV dec^−1^ and a small overpotential of 252 mV at 10 mA cm^−2^ (Fig. 14d). This outstanding catalytic performance and stability for OER are ascribed to the following superiorities. The large porous structure provides more accessible surface to contact with electrolytes. More exposed active edges facilitate the transfer process of the proton-coupled electrons during the OER process. In addition, the CoAl LDH nanosheets firmly covered on 3DGN could improve the electron/charge transfer and reaction kinetics and prevent the adverse aggregating of nanosheets. Qiao et al. incorporated NiCo LDH into N-doped graphene hydrogels (NG-NiCo) to fabricate a 3D architecture as electrocatalysts (Fig. 14e–h) [113]. The numerous functional groups on the functionalized graphene can promote the adsorption of the reaction intermediates. The in situ growth of NiCo LDH ensures the reduced contact resistance between NiCo LDH and graphene, and the 3D interconnected porous network offers more exposed active sites and favors the O_2_ release. Combining with the multiple advantages of 3D N-doped graphene hydrogels, the NG-NiCo exhibits favorable electrode kinetics, superior activity, and great durability (Fig. 14i–k).

**Fig. 15:**
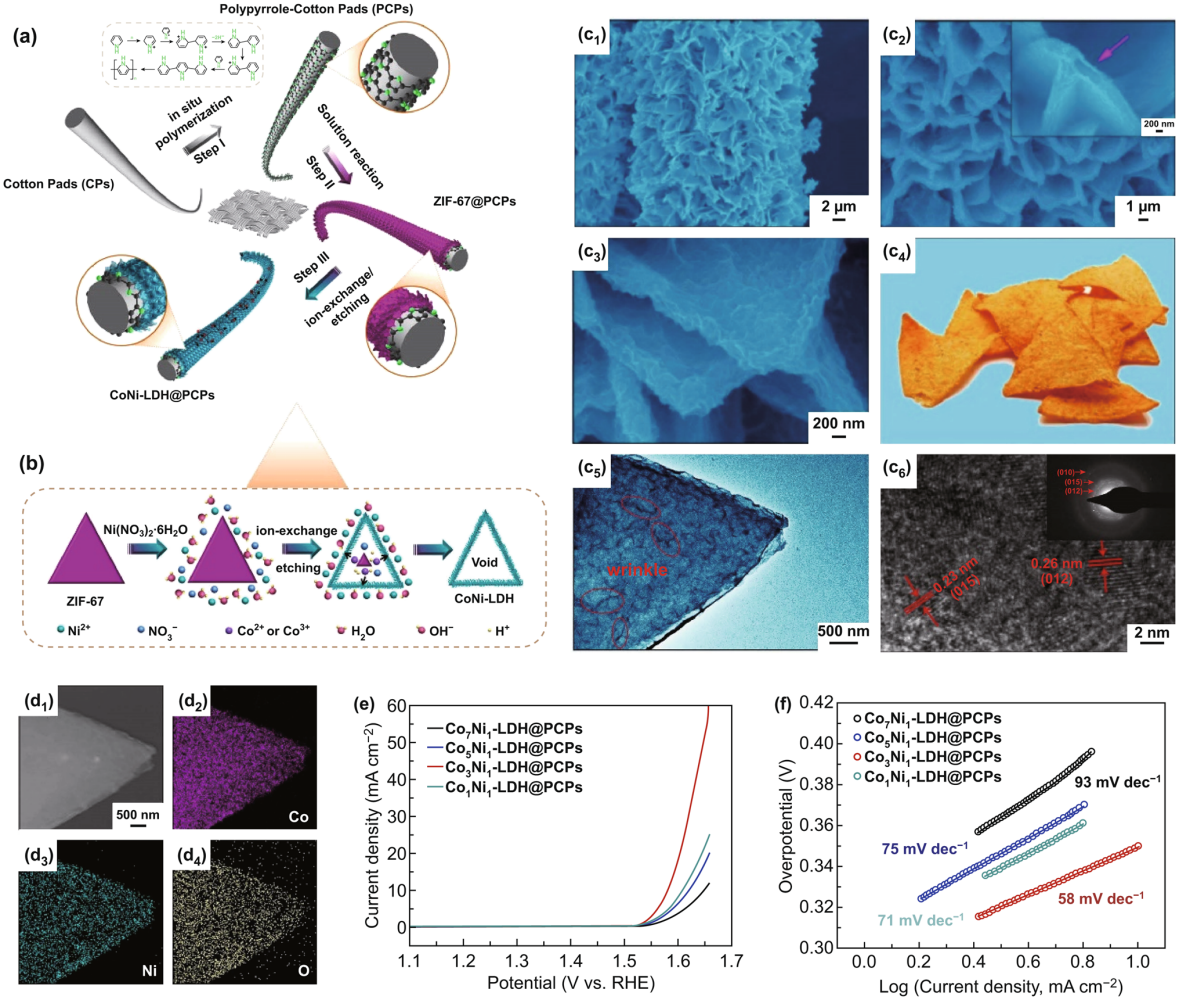
**a** Schematic diagram of the preparation process of CoNi-LDH@PCPs. **b** Formation mechanism of hollow CoNi-LDH arrays. **c** SEM images (1–3) and HRTEM images (5–6) of CoNi-LDH@PCPs, and photograph (4) of triangle biscuits. **d** STEM image (1) and mapping images of CoNi-LDH arrays. **e** LSV curves of the different CoNi-LDH@PCPs catalysts at 1 mV s^−1^ in 1 M KOH. **f** Tafel plots of the different CoNi-LDH@PCPs catalysts. Reproduced with permission of Ref. [124]. Copyright 2019 American Chemical Society

Recently, some highly conductive 3D networks (e.g., carbon paper/cloth/foam, carbon nanotube/graphene film, and Ni/Fe/Cu foam) are used as the current collector to in situ fabricate binder-free LDH-based electrodes for OER [133, 134]. Chen et al. fabricated a microfiber electrode by incorporating aligned carbon nanotubes with NiFe LDH nanoparticles [133]. This microfiber electrode shows high electrocatalytic surface area and strengthened contact between electrocatalysts and substrate, which also avoids using binders (*e.g.,* Nafion and polytetrafluoroethylene), thus resulting in the outstanding durability and remarkable OER activity with a low overpotential of 255 mV at 180 mA cm^−2^. Likewise, Song et al. developed an integrated flexible electrode by coupling NiFe LDH with single-walled carbon nanotubes (SWNT) film via a facile hydrothermal method [134]. The NiFe LDH@SWNT electrode shows the fast reaction kinetics with a Tafel slope of 35 mV dec^−1^, together with an excellent OER activity with a low overpotential of 250 mV at 10 mA cm^−2^. As mentioned above, the superior OER performance is authentically contributed to the strong interfacial electron coupling between the highly active LDH and the conductive support (SWNT). Yu et al. successfully synthesized NiCo LDH nanoarrays vertically grown on carbon fiber papers (NiCo LDH-NA) by a hydrothermal method [135]. Compared with NiCo LDH microspheres (NiCo LDH-MS), the NiCo LDH-NA catalyst has a low Tafel slope of 64 mV decade^−1^ and needs a relatively low overpotential of 307 mV to obtain 10 mA cm^−2^. This high OER activity profits from the synergistic effect of the highly conductive substrate (carbon fiber paper) and vertically oriented LDH nanoarrays with abundant rich active sites, open structure, and high surface areas. Yu et al. synthesized 3D core–shell NiFe LDHs@Cu nanowires grown on Cu foam (Cu@NiFe LDH/CF) through chemical oxidation followed by calcination and electronic reduction [136]. The Cu nanowires are uniformly coated with few-layer NiFe LDH nanosheets to form a typical core–shell structure. The obtained 3D core–shell electrode shows the distinctly boosted OER activity, which can be attributed to the following fundamental factors. The 3D Cu nanowires network with high conductivity ensures fast electron transfer and ions diffusion, and the firm adhesion between LDHs and Cu nanowires grown on Cu foam is conducive to obtaining the high mechanical stability and good electrical contacts without using binders. The vertically grown LDH nanosheets with enlarged active surface afford more efficient catalytic sites for OER. As for carbon-based or metal-based conductive substrates, the weak hydrophilicity, large density, and poor flexibility limit their practicability. Luo et al. developed a new hollow potato chip-like CoNi-LDH@polypyrrole cotton pads (CoNi-LDH@PCPs) catalytic electrode [124]. Figure 15a, b depicts the synthetic process involving three major steps as follows. Firstly, the polypyrrole-coated cotton pads are prepared by in situ polymerization reaction involving chemical oxidation and electrochemical initiation. Subsequently, the as-prepared PCPs are used as the skeleton to grow ZIF-67 arrays through a facile solution reaction. Finally, the hollow structural CoNi-LDH arrays on PCPs are formed after the ion exchange/etching process. It can be found that the flake-like CoNi-LDH arrays are vertically aligned on the PCPs’ surface (Fig. 15c, d). The hollow cavities of the CoNi-LDH arrays can be confirmed from the fragmented part (Fig. 15c_2_). As shown in Fig. 15c_3_, c_4_, the CoNi-LDH arrays with a quite rough surface show a potato chip-like structure. Due to the existence of the hollow potato chip-like structure, reaction kinetics on the electrode can be effectively expedited. Furthermore, the 3D PCPs framework offers the efficient transport pathways for electrons/ions, and larger open space formed by the adjacent fibers is beneficial to the electrolyte infiltration and bubble evolution. As an OER catalyst, this CoNi-LDH@PCPs electrode exhibits a low overpotential of 350 mV at 10 mA cm^−2^ and a small Tafel slope of ∼ 58 mV dec^−1^ (Fig. 15e, f).

**Fig. 16:**
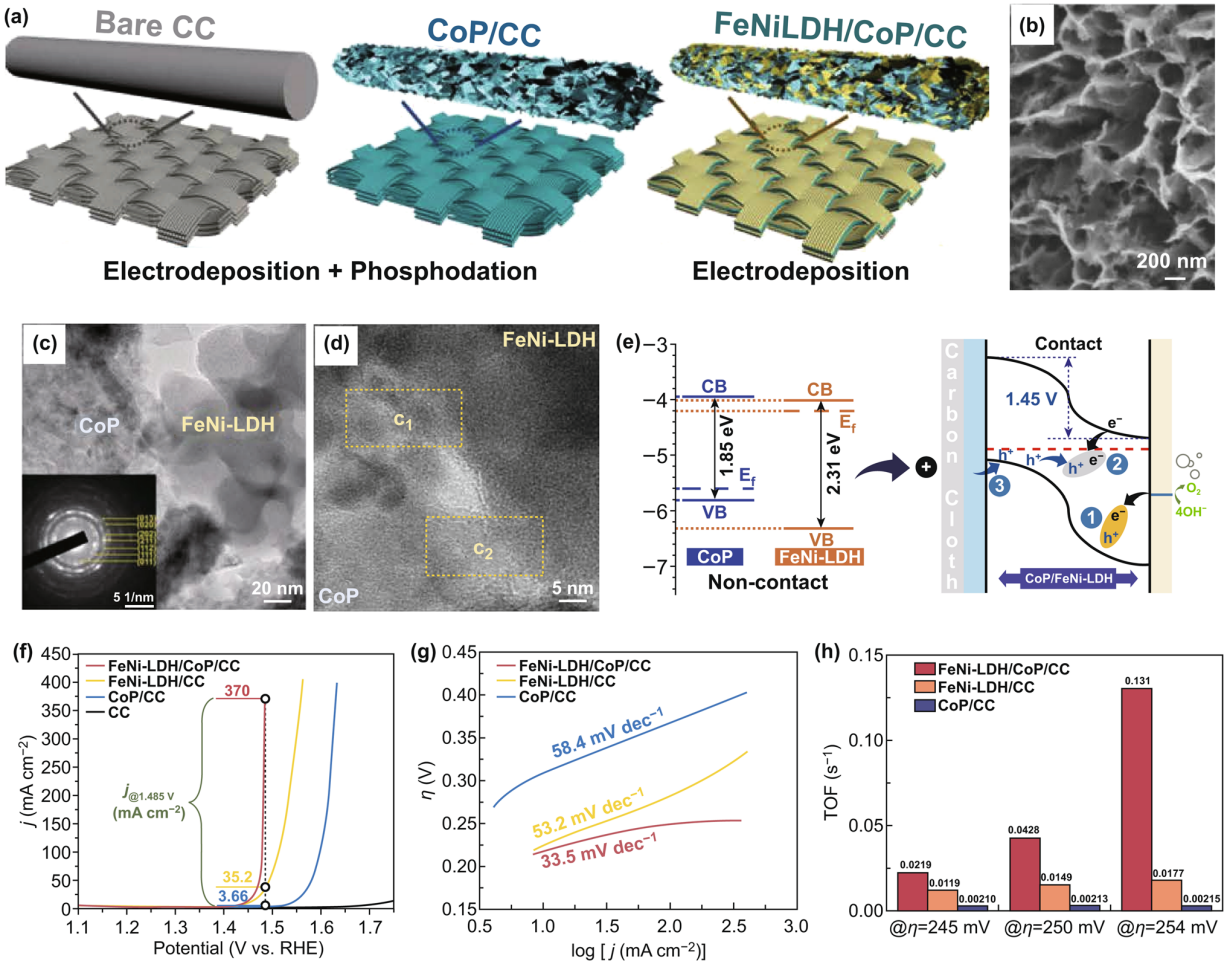
**a** Schematic diagram of the fabrication process of FeNi-LDH/CoP/CC composite electrodes. **b** SEM image, **c**, **d** HRTEM images and SAED pattern (inset of **c**) of FeNi-LDH/CoP NSs. **e** The energy diagrams of CoP and FeNi-LDH and the electrocatalytic mechanism for OER in the FeNi-LDH/CoP/CC p–n junction. **f** LSV curves of the different CoNi-LDH@PCPs catalysts at 5 mV s^−1^ in 1 M KOH. **g** Tafel plots of the different catalysts. **h** TOF at different overpotentials. Reproduced with permission of Ref. [125]. Copyright 2019 Wiley–VCH Verlag GmbH & Co. KGaA, Weinheim

Recently, LDHs have been combined with conductive metallic substrates to construct 3D hierarchical hybrid arrays. The plentiful catalytic sites from multiple species and 3D channels are beneficial to further enhance the catalytic performance of the LDH/conductive support catalysts toward OER. Yuan et al. developed a 3D hierarchical CoFe-LDH@NiFe LDH hybrid nanosheet array on nickel foam (CoFe@NiFe/NF) by a facile hydrothermal process followed by the electrodeposition method [137]. This CoFe@NiFe/NF catalyst shows high activity and stability for both OER and HER, owing to its unique structural features and strong synergistic effect between two kinds of LDHs. When the CoFe@NiFe/NF is used as both cathode and anode in an alkali electrolyte, it needs a low voltage of 1.59 V to achieve 10 mA cm^−2^, which is much lower than many other state-of-the-art earth-abundant catalysts. Metal phosphides have been regarded as a promising candidate because of their excellent catalytic activity and metalloid characteristics. Zhou et al. reported ultrathin NiCoP/NiFe LDH nanosheet arrays supported on nickel foam, which can be served as a high-efficiency OER catalyst [138]. Besides the advantages of this 3D hierarchical binder-free structure, the abundant multimetallic catalytic sites of NiCoP and NiFe LDH promote the OER activity. Qian et al. proposed a concept for promoting the catalytic activities for OER by constructing the FeNi-LDH/CoP/carbon cloth (CC) heterojunctions [125]. The self-supporting FeNi-LDH/CoP/CC electrode with an open and 3D hierarchical p–n junction structure is prepared through three steps including the electrodeposition of Co(OH)_2_ nanosheets array on the CC, phosphatization of Co(OH)_2_, and electrodeposition of amorphous FeNi-LDH layers on the formed CoP array (Fig. 16a–d). The charge transfer and separation at the interfaces of FeNi-LDH/CoP p–n junctions result in positively charged FeNi-LDH side. OH^−^ ions intend to adsorb on the surface of FeNi-LDH side in the p–n junction more strongly compared to individual FeNi-LDH as verified by DFT calculation, which indicates that this positively charged FeNi-LDH side has stronger ability to adsorb targeted OH^-^ compared with individual FeNi-LDH (Fig. 16e). As a result, the FeNi-LDH/CoP/CC electrode has the low overpotentials of 231, 249, and 254 mV at 20, 100, and 350 mA cm^−2^ in alkaline media, respectively, and its current density at 1.485 V increases by 10 times and 100 times compared to the FeNi-LDH/CC and CoP/CC, respectively. In addition, an extremely small Tafel slope (33.5 mV dec^−1^) and a large TOF (0.131 s^−1^) can be obtained (Fig. 16f–h). The fabrication of heterojunctions in catalyst would be a new strategy to promote their catalytic activities by purposefully regulating the electronic structure of active sites. For the past few years, many other 3D hierarchical nanoarrays on conductive 3D networks, such as CoFe LDH/Co_0.85_Se/carbon cloth, NiFe LDH@NiFe-Bi/carbon cloth, and NiSe/NiFe LDH/Ni foam, have been reported as high-performance OER catalysts [139–144].

## Summary and Outlook

Electrochemical water oxidation is a critical process of water splitting which exhibits a great potential for energy storage and conversion. Designing and synthesizing low-cost highly active electrocatalysts are essential to improve the efficiency for applications on an industrial scale. In this review, we summarized recent notable developments of LDH nanosheets and their derivates toward OER electrocatalysis, together with important strategies to enhance their electrocatalytic activities. First, various synthetic procedures to control the morphology and phases are presented, covering bottom-up and top-down approaches. Secondly, in order to enhance the intrinsic activity of LDHs, several typical approaches have been introduced, including doping other metal or nonmetal components or creating cavities. Moreover, considering the poor conductivity of LDHs, it becomes very beneficial to exfoliate them into monolayer or few layers and then hybridizing with conductive components. In situ growing LDH nanosheets on conductive substrates to fabricate 3D freestanding electrodes have also proven to be an effective methodology to promoting their intrinsic activity.

It is anticipated that the design and synthesis of new composites based on LDHs with controllable structure and morphology for electrochemical water splitting will be the future direction. The remaining challenge is to elaborately tune the electronic structure and control the quantities of active sites in LDHs. It is also needed to probe the electrocatalytic process in situ and elucidate the mechanism in depth, thus providing clear guidance for the rational design of LDHs and their derivates as next-generation nonprecious electrocatalysts. It is also noteworthy that the applications of transition metal LDHs may be broadened to other related energy storage fields such as supercapacitors and batteries. Nevertheless, enormous challenges still exist in achieving practical electrochemical water oxidation using these nanocatalysts. Future efforts should be directed toward making full use of the structural superiority of LDHs and probing the fundamental principles, such as systematically investigating the versatile combination of metal cationic species and valence states, modulating anionic gallery as well as tuning interlayer spacing. The catalytic properties under large current conditions and long-term stability have yet to meet the requirements of implementation on any industrial scale. Much more solid works are urgently needed to address these issues.

## References

[1] Burke MS, Enman LJ, Batchellor AS, Zou S, Boettcher SW (2015). Oxygen evolution reaction electrocatalysis on transition metal oxides and (oxy)hydroxides: activity trends and design principles. Chem. Mater..

[2] Suntivich J, May KJ, Gasteiger HA, Goodenough JB, Shao-Horn Y (2011). A perovskite oxide optimized for oxygen evolution catalysis from molecular orbital principles. Science.

[3] Zhao S, Wang Y, Dong J, He C-T, Yin H (2016). Ultrathin metal–organic framework nanosheets for electrocatalytic oxygen evolution. Nat. Energy.

[4] Meng Y, Zhang X, Hung W-H, He J, Tsai Y-S (2019). Highly active oxygen evolution integrated with efficient CO_2_ to CO electroreduction. Proc. Natl. Acad. Sci. U.S.A..

[5] Zeradjanin AR (2018). Is a major breakthrough in the oxygen electrocatalysis possible?. Curr. Opin. Electrochem..

[6] Seitz LC, Dickens CF, Nishio K, Hikita Y, Montoya J (2016). A highly active and stable IrOx/SrIrO_3_ catalyst for the oxygen evolution reaction. Science.

[7] Lu Z, Xu W, Zhu W, Yang Q, Lei X (2014). Three-dimensional NiFe layered double hydroxide film for high-efficiency oxygen evolution reaction. Chem. Commun..

[8] Tahir M, Pan L, Idrees F, Zhang X, Wang L, Zou J-J, Wang ZL (2017). Electrocatalytic oxygen evolution reaction for energy conversion and storage: a comprehensive review. Nano Energy.

[9] Lu X, Hao G-P, Sun X, Kaskel S, Schmidt OG (2017). Highly dispersed metal and oxide nanoparticles on ultra-polar carbon as efficient cathode materials for Li–O_2_ batteries. J. Mater. Chem. A.

[10] Lu X, Zheng L, Zhang M, Tang H, Li X, Liao S (2017). Synthesis of core-shell structured Ru@Pd/C catalysts for the electrooxidation of formic acid. Electrochim. Acta.

[11] Lv L, Yang Z, Chen K, Wang C, Xiong Y (2019). 2d layered double hydroxides for oxygen evolution reaction: from fundamental design to application. Adv. Energy Mater..

[12] Song F, Hu X (2014). Exfoliation of layered double hydroxides for enhanced oxygen evolution catalysis. Nat. Commun..

[13] Han N, Zhao F, Li Y (2015). Ultrathin nickel–iron layered double hydroxide nanosheets intercalated with molybdate anions for electrocatalytic water oxidation. J. Mater. Chem. A.

[14] Wang Y, Yan D, El Hankari S, Zou Y, Wang S (2018). Recent progress on layered double hydroxides and their derivatives for electrocatalytic water splitting. Adv. Sci..

[15] Yu J, Wang Q, O’Hare D, Sun L (2017). Preparation of two dimensional layered double hydroxide nanosheets and their applications. Chem. Soc. Rev..

[16] Liu Y, Wang N, Pan JH, Steinbach F, Caro J (2014). In situ synthesis of MOF membranes on ZnAl–CO_3_ LDH buffer layer-modified substrates. J. Am. Chem. Soc..

[17] Ma R, Liang J, Liu X, Sasaki T (2012). General insights into structural evolution of layered double hydroxide: underlying aspects in topochemical transformation from brucite to layered double hydroxide. J. Am. Chem. Soc..

[18] Zhitova ES, Krivovichev SV, Pekov IV, Yapaskurt VO (2019). Crystal chemistry of chlormagaluminite, Mg_4_A_l2_(OH)_12_Cl_2_(H_2_O)_2_, a natural layered double hydroxide. Minerals.

[19] Cai Z, Bu X, Wang P, Ho JC, Yang J, Wang X (2019). Recent advances in layered double hydroxide electrocatalysts for the oxygen evolution reaction. J. Mater. Chem. A.

[20] Huang L, Zou Y, Chen D, Wang S (2019). Electronic structure regulation on layered double hydroxides for oxygen evolution reaction. Chin. J. Catal..

[21] Liu Z, Dong C-L, Huang Y-C, Cen J, Yang H (2019). Modulating the electronic structure of ultrathin layered double hydroxide nanosheets with fluorine: an efficient electrocatalyst for the oxygen evolution reaction. J. Mater. Chem. A.

[22] Ma R, Liang J, Takada K, Sasaki T (2010). Topochemical synthesis of Co–Fe layered double hydroxides at varied Fe/Co ratios: unique intercalation of triiodide and its profound effect. J. Am. Chem. Soc..

[23] Suen N-T, Hung S-F, Quan Q, Zhang N, Xu Y-J, Chen HM (2017). Electrocatalysis for the oxygen evolution reaction: recent development and future perspectives. Chem. Soc. Rev..

[24] Lu X, Si W, Sun X, Liu B, Zhang L, Yan C, Schmidt OG (2016). Pd-functionalized MnOx–GeOy nanomembranes as highly efficient cathode materials for Li–O_2_ batteries. Nano Energy.

[25] Zhang H (2015). Ultrathin two-dimensional nanomaterials. ACS Nano.

[26] Lu X, Yin Y, Zhang L, Huang S, Xi L, Liu L, Oswald S, Schmidt OG (2019). 3d Ag/NiO–Fe_2_O_3_/Ag nanomembranes as carbon-free cathode materials for Li–O_2_ batteries. Energy Storage Mater..

[27] Hur T-B, Phuoc TX, Chyu MK (2010). New approach to the synthesis of layered double hydroxides and associated ultrathin nanosheets in de-ionized water by laser ablation. J. Appl. Phys..

[28] Phuoc TX, Soong Y, Chyu MK (2007). Synthesis of Ag-deionized water nanofluids using multi-beam laser ablation in liquids. Opt. Lasers Eng..

[29] Sasaki T, Liang C, Nichols WT, Shimizu Y, Koshizaki N (2004). Fabrication of oxide base nanostructures using pulsed laser ablation in aqueous solutions. Appl. Phys. A.

[30] Kazakevich PV, Simakin AV, Voronov VV, Shafeev GA (2006). Laser induced synthesis of nanoparticles in liquids. Appl. Surf. Sci..

[31] Compagnini G, Messina E, Puglisi O, Cataliotti RS, Nicolosi V (2008). Spectroscopic evidence of a core–shell structure in the earlier formation stages of Au–Ag nanoparticles by pulsed laser ablation in water. Chem. Phys. Lett..

[32] Hunter BM, Blakemore JD, Deimund M, Gray HB, Winkler JR, Müller AM (2014). Highly active mixed-metal nanosheet water oxidation catalysts made by pulsed-laser ablation in liquids. J. Am. Chem. Soc..

[33] Qiao C, Zhang Y, Zhu Y, Cao C, Bao X, Xu J (2015). One-step synthesis of Zinc–Cobalt layered double hydroxide (Zn–Co-LDH) nanosheets for high-efficiency oxygen evolution reaction. J. Mater. Chem. A.

[34] Yan Y, Liu Q, Wang J, Wei J, Gao Z (2012). Single-step synthesis of layered double hydroxides ultrathin nanosheets. J. Colloid Interface Sci..

[35] Hu G, Wang N, O’Hare D, Davis J (2006). One-step synthesis and AFM imaging of hydrophobic LDH monolayers. Chem. Commun..

[36] Reichle WT (1986). Synthesis of anionic clay minerals (mixed metal hydroxides, hydrotalcite). Solid State Ionics.

[37] Ehlsissen KT, Delahaye-Vidal A, Genin P, Figlarz M, Willmann P (1993). Preparation and characterization of turbostratic Ni/Al layered double hydroxides for nickel hydroxide electrode applications. J. Mater. Chem..

[38] Xu R, Zeng HC (2001). Synthesis of nanosize supported hydrotalcite-like compounds CoAl_*x*_(OH)_2+2*x*_(CO_3_)_*y*_(NO_3_)_*x*-2*y*_*·n*H_2_O on γ-Al_2_O_3_. Chem. Mater..

[39] Nobuo I, Taki M, Yoshiro K, Kenji K (2004). A novel synthetic route to layered double hydroxides using hexamethylenetetramine. Chem. Lett..

[40] Cai H, Hillier AC, Franklin KR, Nunn CC, Ward MD (1994). Nanoscale imaging of molecular adsorption. Science.

[41] Bitsianes G, Joseph TL (1955). Topochemical aspects of iron ore reduction. JOM.

[42] Ma R, Liu Z, Takada K, Iyi N, Bando Y, Sasaki T (2007). Synthesis and exfoliation of Co^2+^−Fe^3+^ layered double hydroxides: an innovative topochemical approach. J. Am. Chem. Soc..

[43] Ma R, Takada K, Fukuda K, Iyi N, Bando Y, Sasaki T (2008). Topochemical synthesis of monometallic (Co^2+^–Co^3+^) layered double hydroxide and its exfoliation into positively charged Co(OH)_2_ nanosheets. Angew. Chem. Int. Ed..

[44] Lee J-H, O’Hare D, Jung D-Y (2012). Topochemical oxidation of transition metals in layered double hydroxides by anthraquinone-2-sulfonate. Bull. Korean Chem. Soc..

[45] Lee JH, Du Y, O’Hare D (2009). Growth of oriented thin films of intercalated α-cobalt hydroxide on functionalized Au and Si substrates. Chem. Mater..

[46] Ma L, Wang Q, Islam SM, Liu Y, Ma S, Kanatzidis MG (2016). Highly selective and efficient removal of heavy metals by layered double hydroxide intercalated with the MoS_4_^2–^ ion. J. Am. Chem. Soc..

[47] Wang D-Y, Costa FR, Vyalikh A, Leuteritz A, Scheler U (2009). One-step synthesis of organic LDH and its comparison with regeneration and anion exchange method. Chem. Mater..

[48] Sene S, Bégu S, Gervais C, Renaudin G, Mesbah A (2015). Intercalation of benzoxaborolate anions in layered double hydroxides: toward hybrid formulations for benzoxaborole drugs. Chem. Mater..

[49] Ma L, Islam SM, Liu H, Zhao J, Sun G, Li H, Ma S, Kanatzidis MG (2017). Selective and efficient removal of toxic oxoanions of As(iii), As(v), and Cr(vi) by layered double hydroxide intercalated with MoS_4_^2–^. Chem. Mater..

[50] Lv L, Xu K, Wang C, Wan H, Ruan Y (2016). Intercalation of glucose in NiMn-layered double hydroxide nanosheets: an effective path way towards battery-type electrodes with enhanced performance. Electrochim. Acta.

[51] Liu W, Xu S, Liang R, Wei M, Evans DG, Duan X (2017). In situ synthesis of nitrogen-doped carbon dots in the interlayer region of a layered double hydroxide with tunable quantum yield. J. Mater. Chem. C.

[52] Wang C, Zhang X, Sun X, Ma Y (2016). Facile fabrication of ethylene glycol intercalated cobalt-nickel layered double hydroxide nanosheets supported on nickel foam as flexible binder-free electrodes for advanced electrochemical energy storage. Electrochim. Acta.

[53] Liu Z, Ma R, Osada M, Iyi N, Ebina Y, Takada K, Sasaki T (2006). Synthesis, anion exchange, and delamination of Co–Al layered double hydroxide: assembly of the exfoliated nanosheet/polyanion composite films and magneto-optical studies. J. Am. Chem. Soc..

[54] Adachi-Pagano M, Forano C, Besse J-P (2000). Delamination of layered double hydroxides by use of surfactants. Chem. Commun..

[55] Venugopal BR, Shivakumara C, Rajamathi M (2006). Effect of various factors influencing the delamination behavior of surfactant intercalated layered double hydroxides. J. Colloid Interface Sci..

[56] Naik VV, Ramesh TN, Vasudevan S (2011). Neutral nanosheets that gel: exfoliated layered double hydroxides in toluene. J. Phys. Chem. Lett..

[57] Liang J, Ma R, Iyi N, Ebina Y, Takada K, Sasaki T (2010). Topochemical synthesis, anion exchange, and exfoliation of Co–Ni layered double hydroxides: a route to positively charged Co–Ni hydroxide nanosheets with tunable composition. Chem. Mater..

[58] Hibino T, Jones W (2001). New approach to the delamination of layered double hydroxides. J. Mater. Chem..

[59] Hibino T (2004). Delamination of layered double hydroxides containing amino acids. Chem. Mater..

[60] Iyi N, Sasaki T (2008). Decarbonation of MgAl-LDHs (layered double hydroxides) using acetate–buffer/NaCl mixed solution. J. Colloid Interface Sci..

[61] Li L, Ma R, Ebina Y, Iyi N, Sasaki T (2005). Positively charged nanosheets derived via total delamination of layered double hydroxides. Chem. Mater..

[62] Iyi N, Ishihara S, Kaneko Y, Yamada H (2013). Swelling and gel/sol formation of perchlorate-type layered double hydroxides in concentrated aqueous solutions of amino acid-related zwitterionic compounds. Langmuir.

[63] Langmuir I (1928). Oscillations in ionized gases. Proc. Natl. Acad. Sci..

[64] Shim J, Oh A, Kang D-H, Oh S, Jang SK (2016). High-performance 2d rhenium disulfide (ReS_2_) transistors and photodetectors by oxygen plasma treatment. Adv. Mater..

[65] Wang C, Zhou Y, He L, Ng T-W, Hong G (2013). In situ nitrogen-doped graphene grown from polydimethylsiloxane by plasma enhanced chemical vapor deposition. Nanoscale.

[66] Girshick SL, Chiu C-P (1989). Homogeneous nucleation of particles from the vapor phase in thermal plasma synthesis. Plasma Chem. Plasma Process..

[67] Moshrefi MM, Rashidi F (2018). Hydrogen production from methane decomposition in cold plasma reactor with rotating electrodes. Plasma Chem. Plasma Process..

[68] Mangolini L, Thimsen E, Kortshagen U (2005). High-yield plasma synthesis of luminescent silicon nanocrystals. Nano Lett..

[69] Chu PK, Chen JY, Wang LP, Huang N (2002). Plasma-surface modification of biomaterials. Mater. Sci. Eng., R.

[70] Tao L, Lin C-Y, Dou S, Feng S, Chen D (2017). Creating coordinatively unsaturated metal sites in metal-organic-frameworks as efficient electrocatalysts for the oxygen evolution reaction: insights into the active centers. Nano Energy.

[71] Zhang Y, Ouyang B, Xu J, Jia G, Chen S, Rawat RS, Fan HJ (2016). Rapid synthesis of cobalt nitride nanowires: highly efficient and low-cost catalysts for oxygen evolution. Angew. Chem. Int. Ed..

[72] Wang K, Xu M, Gu Y, Gu Z, Liu J, Fan QH (2017). Low-temperature plasma exfoliated N-doped graphene for symmetrical electrode supercapacitors. Nano Energy.

[73] Lu W, Nan H, Hong J, Chen Y, Zhu C (2014). Plasma-assisted fabrication of monolayer phosphorene and its Raman characterization. Nano Res..

[74] Wang Y, Zhang Y, Liu Z, Xie C, Feng S, Liu D, Shao M, Wang S (2017). Layered double hydroxide nanosheets with multiple vacancies obtained by dry exfoliation as highly efficient oxygen evolution electrocatalysts. Angew. Chem. Int. Ed..

[75] Liu R, Wang Y, Liu D, Zou Y, Wang S (2017). Water-plasma-enabled exfoliation of ultrathin layered double hydroxide nanosheets with multivacancies for water oxidation. Adv. Mater..

[76] Wang Y, Xie C, Zhang Z, Liu D, Chen R, Wang S (2018). In situ exfoliated, N-doped, and edge-rich ultrathin layered double hydroxides nanosheets for oxygen evolution reaction. Adv. Funct. Mater..

[77] Zhou D, Wang S, Jia Y, Xiong X, Yang H (2019). Nife hydroxide lattice tensile strain: enhancement of adsorption of oxygenated intermediates for efficient water oxidation catalysis. Angew. Chem. Int. Ed..

[78] Vialat P, Mousty C, Taviot-Gueho C, Renaudin G, Martinez H, Dupin J-C, Elkaim E, Leroux F (2014). High-performing monometallic cobalt layered double hydroxide supercapacitor with defined local structure. Adv. Funct. Mater..

[79] Parveen N, Cho MH (2016). Self-assembled 3d flower-like nickel hydroxide nanostructures and their supercapacitor applications. Sci. Rep..

[80] Salunkhe RR, Lin J, Malgras V, Dou SX, Kim JH, Yamauchi Y (2015). Large-scale synthesis of coaxial carbon nanotube/Ni(OH)_2_ composites for asymmetric supercapacitor application. Nano Energy.

[81] Wang J, Ni Y, Jiang W, Li H, Liu Y, Lin S, Zhou Y, Yan D (2015). Self-crosslinking and surface-engineered polymer vesicles. Small.

[82] Jiang J, Sun F, Zhou S, Hu W, Zhang H (2018). Atomic-level insight into super-efficient electrocatalytic oxygen evolution on iron and vanadium co-doped nickel (oxy)hydroxide. Nat. Commun..

[83] Cao L-M, Wang J-W, Zhong D-C, Lu T-B (2018). Template-directed synthesis of sulphur doped NiCoFe layered double hydroxide porous nanosheets with enhanced electrocatalytic activity for the oxygen evolution reaction. J. Mater. Chem. A.

[84] Bates MK, Jia Q, Doan H, Liang W, Mukerjee S (2016). Charge-transfer effects in Ni–Fe and Ni–Fe–Co mixed-metal oxides for the alkaline oxygen evolution reaction. ACS Catal..

[85] Lu Z, Qian L, Tian Y, Li Y, Sun X, Duan X (2016). Ternary NiFeMn layered double hydroxides as highly-efficient oxygen evolution catalysts. Chem. Commun..

[86] Lado JL, Wang X, Paz E, Carbó-Argibay E, Guldris N (2015). Design and synthesis of highly active Al–Ni–P foam electrode for hydrogen evolution reaction. ACS Catal..

[87] Tang C, Zhang R, Lu W, He L, Jiang X, Asiri AM, Sun X (2017). Fe-doped CoP nanoarray: a monolithic multifunctional catalyst for highly efficient hydrogen generation. Adv. Mater..

[88] Zhou P, He J, Zou Y, Wang Y, Xie C, Chen R, Zang S, Wang S (2019). Single-crystalline layered double hydroxides with rich defects and hierarchical structure by mild reduction for enhancing the oxygen evolution reaction. Sci. China Chem..

[89] Wang Y, Qiao M, Li Y, Wang S (2018). Tuning surface electronic configuration of NiFe LDHs nanosheets by introducing cation vacancies (Fe or Ni) as highly efficient electrocatalysts for oxygen evolution reaction. Small.

[90] Mignani A, Ballarin B, Giorgetti M, Scavetta E, Tonelli D (2013). Heterostructure of Au nanoparticles—NiAl layered double hydroxide: electrosynthesis, characterization, and electrocatalytic properties. J. Phys. Chem. C.

[91] Xu L, Qu Z, Chen J, Chen X, Li F, Yang W (2017). Highly dispersed palladium nanoparticles generated in situ on layered double hydroxide nanowalls for ultrasensitive electrochemical detection of hydrazine. Anal. Methods.

[92] Zhu W, Liu L, Yue Z, Zhang W, Yue X (2017). Au promoted nickel–iron layered double hydroxide nanoarrays: a modular catalyst enabling high-performance oxygen evolution. ACS Appl. Mater. Interfaces.

[93] Gao X, Long X, Yu H, Pan X, Yi Z (2017). Ni nanoparticles decorated NiFe layered double hydroxide as bifunctional electrochemical catalyst. J. Electrochem. Soc..

[94] Deng X, Huang J, Wan H, Chen F, Lin Y, Xu X, Ma R, Sasaki T (2019). Recent progress in functionalized layered double hydroxides and their application in efficient electrocatalytic water oxidation. J. Energy Chem..

[95] Qiao B, Wang A, Yang X, Allard LF, Jiang Z (2011). Single-atom catalysis of CO oxidation using Pt1/FeOx. Nat. Chem..

[96] Liu P, Zhao Y, Qin R, Mo S, Chen G (2016). Photochemical route for synthesizing atomically dispersed palladium catalysts. Science.

[97] Zhang J, Liu J, Xi L, Yu Y, Chen N (2018). Single-atom Au/NiFe layered double hydroxide electrocatalyst: probing the origin of activity for oxygen evolution reaction. J. Am. Chem. Soc..

[98] Zhang B, Zhu C, Wu Z, Stavitski E, Lui YH (2020). Integrating rh species with NiFe-layered double hydroxide for overall water splitting. Nano Lett..

[99] Wang Z, Xu S-M, Xu Y, Tan L, Wang X, Zhao Y, Duan H, Song Y-F (2019). Single Ru atoms with precise coordination on a monolayer layered double hydroxide for efficient electrooxidation catalysis. Chem. Sci..

[100] Valdez R, Grotjahn DB, Smith DK, Quintana JM, Olivas A (2015). Nanosheets of Co-(Ni and Fe) layered double hydroxides for electrocatalytic water oxidation reaction. Int. J. Electrochem. Sci..

[101] Tang D, Liu J, Wu X, Liu R, Han X (2014). Carbon quantum dot/NiFe layered double-hydroxide composite as a highly efficient electrocatalyst for water oxidation. ACS Appl. Mater. Interfaces.

[102] Baker SN, Baker GA (2010). Luminescent carbon nanodots: emergent nanolights. Angew. Chem. Int. Ed..

[103] Li H, He X, Kang Z, Huang H, Liu Y (2010). Water-soluble fluorescent carbon quantum dots and photocatalyst design. Angew. Chem. Int. Ed..

[104] Sun Y-P, Zhou B, Lin Y, Wang W, Fernando KAS (2006). Quantum-sized carbon dots for bright and colorful photoluminescence. J. Am. Chem. Soc..

[105] Tang C, Wang H-F, Zhu X-L, Li B-Q, Zhang Q (2016). Advances in hybrid electrocatalysts for oxygen evolution reactions: rational integration of NiFe layered double hydroxides and nanocarbon. Part. Part. Syst. Charact..

[106] Wang S, Yu D, Dai L (2011). Polyelectrolyte functionalized carbon nanotubes as efficient metal-free electrocatalysts for oxygen reduction. J. Am. Chem. Soc..

[107] Xu Z, Fan X, Li H, Fu H, Lau WM, Zhao X (2017). Edges of graphene and carbon nanotubes with high catalytic performance for the oxygen reduction reaction. PCCP.

[108] Gong M, Li Y, Wang H, Liang Y, Wu JZ (2013). An advanced Ni–Fe layered double hydroxide electrocatalyst for water oxidation. J. Am. Chem. Soc..

[109] Lee C, Wei X, Kysar JW, Hone J (2008). Measurement of the elastic properties and intrinsic strength of monolayer graphene. Science.

[110] Balandin AA, Ghosh S, Bao W, Calizo I, Teweldebrhan D, Miao F, Lau CN (2008). Superior thermal conductivity of single-layer graphene. Nano Lett..

[111] Long X, Li J, Xiao S, Yan K, Wang Z, Chen H, Yang S (2014). A strongly coupled graphene and FeNi double hydroxide hybrid as an excellent electrocatalyst for the oxygen evolution reaction. Angew. Chem. Int. Ed..

[112] Yi J, Longzhou Z, Guoping G, Hua C, Bei W (2017). A heterostructure coupling of exfoliated Ni–Fe hydroxide nanosheet and defective graphene as a bifunctional electrocatalyst for overall water splitting. Adv. Mater..

[113] Chen S, Duan J, Jaroniec M, Qiao SZ (2013). Three-dimensional N-doped graphene hydrogel/NiCo double hydroxide electrocatalysts for highly efficient oxygen evolution. Angew. Chem. Int. Ed..

[114] Choi CH, Kim M, Kwon HC, Cho SJ, Yun S (2016). Tuning selectivity of electrochemical reactions by atomically dispersed platinum catalyst. Nat. Commun..

[115] Tang C, Wang H-S, Wang H-F, Zhang Q, Tian G-L, Nie J-Q, Wei F (2015). Spatially confined hybridization of nanometer-sized NiFe hydroxides into nitrogen-doped graphene frameworks leading to superior oxygen evolution reactivity. Adv. Mater..

[116] Zhong J-H, Zhang J, Jin X, Liu J-Y, Li Q (2014). Quantitative correlation between defect density and heterogeneous electron transfer rate of single layer graphene. J. Am. Chem. Soc..

[117] Zhou D, Cai Z, Lei X, Tian W, Bi Y (2017). NiCoFe-layered double hydroxides/N-doped graphene oxide array colloid composite as an efficient bifunctional catalyst for oxygen electrocatalytic reactions. Adv. Energy Mater..

[118] Ma W, Ma R, Wang C, Liang J, Liu X, Zhou K, Sasaki T (2015). A superlattice of alternately stacked Ni–Fe hydroxide nanosheets and graphene for efficient splitting of water. ACS Nano.

[119] Liang H, Meng F, Cabán-Acevedo M, Li L, Forticaux A, Xiu L, Wang Z, Jin S (2015). Hydrothermal continuous flow synthesis and exfoliation of NiCo layered double hydroxide nanosheets for enhanced oxygen evolution catalysis. Nano Lett..

[120] Luo M, Cai Z, Wang C, Bi Y, Qian L (2017). Phosphorus oxoanion-intercalated layered double hydroxides for high-performance oxygen evolution. Nano Res..

[121] Li Y, Zhao M, Zhao Y, Song L, Zhang Z (2016). FeNi layered double-hydroxide nanosheets on a 3d carbon network as an efficient electrocatalyst for the oxygen evolution reaction. Part. Part. Syst. Char..

[122] Wang L, Huang X, Xue J (2016). Graphitic mesoporous carbon loaded with iron–nickel hydroxide for superior oxygen evolution reactivity. Chemsuschem.

[123] Ping J, Wang Y, Lu Q, Chen B, Chen J (2016). Self-assembly of single-layer CoAl-layered double hydroxide nanosheets on 3d graphene network used as highly efficient electrocatalyst for oxygen evolution reaction. Adv. Mater..

[124] Wang W, Lu Y, Zhao M, Luo R, Yang Y (2019). Controllable tuning of cobalt nickel-layered double hydroxide arrays as multifunctional electrodes for flexible supercapattery device and oxygen evolution reaction. ACS Nano.

[125] He K, Tadesse Tsega T, Liu X, Zai J, Li X-H (2019). Utilizing the space-charge region of the FeNi-LDH/CoP p–n junction to promote performance in oxygen evolution electrocatalysis. Angew. Chem. Int. Ed..

[126] Ma R, Liu X, Liang J, Bando Y, Sasaki T (2014). Molecular-scale heteroassembly of redoxable hydroxide nanosheets and conductive graphene into superlattice composites for high-performance supercapacitors. Adv. Mater..

[127] Xiong P, Sun B, Sakai N, Ma R, Sasaki T, Wang S, Zhang J, Wang G (2019). 2d superlattices for efficient energy storage and conversion. Adv. Mater..

[128] Islam MS, Kim M, Jin X, Oh SM, Lee N-S, Kim H, Hwang S-J (2018). Bifunctional 2d superlattice electrocatalysts of layered double hydroxide–transition metal dichalcogenide active for overall water splitting. ACS Energy Lett..

[129] Xiong P, Zhang X, Wan H, Wang S, Zhao Y (2019). Interface modulation of two-dimensional superlattices for efficient overall water splitting. Nano Lett..

[130] Yu M, Zhou S, Wang Z, Zhao J, Qiu J (2018). Boosting electrocatalytic oxygen evolution by synergistically coupling layered double hydroxide with mxene. Nano Energy.

[131] Lang X, Hirata A, Fujita T, Chen M (2011). Nanoporous metal/oxide hybrid electrodes for electrochemical supercapacitors. Nat. Nanotechnol..

[132] Lu Z, Yang Q, Zhu W, Chang Z, Liu J, Sun X, Evans DG, Duan X (2012). Hierarchical Co_3_O_4_@Ni-Co-O supercapacitor electrodes with ultrahigh specific capacitance per area. Nano Res..

[133] Chen R, Sun G, Yang C, Zhang L, Miao J (2016). Achieving stable and efficient water oxidation by incorporating NiFe layered double hydroxide nanoparticles into aligned carbon nanotubes. Nanoscale Horizons.

[134] Liu H, Zhou J, Wu C, Wang C, Zhang Y (2018). Integrated flexible electrode for oxygen evolution reaction: layered double hydroxide coupled with single-walled carbon nanotubes film. ACS Sustain. Chem. Eng..

[135] Yu C, Liu Z, Han X, Huang H, Zhao C, Yang J, Qiu J (2016). NiCo-layered double hydroxides vertically assembled on carbon fiber papers as binder-free high-active electrocatalysts for water oxidation. Carbon.

[136] Yu L, Zhou H, Sun J, Qin F, Yu F (2017). Cu nanowires shelled with NiFe layered double hydroxide nanosheets as bifunctional electrocatalysts for overall water splitting. Energy Environ. Sci..

[137] Yang R, Zhou Y, Xing Y, Li D, Jiang D, Chen M, Shi W, Yuan S (2019). Synergistic coupling of CoFe-LDH arrays with NiFe-LDH nanosheet for highly efficient overall water splitting in alkaline media. Appl. Catal. B.

[138] Zhou L, Jiang S, Liu Y, Shao M, Wei M, Duan X (2018). Ultrathin CoNiP@layered double hydroxides core–shell nanosheets arrays for largely enhanced overall water splitting. ACS Appl. Energy Mater..

[139] Jin W, Liu F, Guo X, Zhang J, Zheng L (2019). Self-supported CoFe LDH/Co_0.85_Se nanosheet arrays as efficient electrocatalysts for the oxygen evolution reaction. Catal. Sci. Technol..

[140] Zhou J, Yu L, Zhu Q, Huang C, Yu Y (2019). Defective and ultrathin nife LDH nanosheets decorated on V-doped Ni_3_S_2_ nanorod arrays: a 3d core–shell electrocatalyst for efficient water oxidation. J. Mater. Chem. A.

[141] Kong F, Zhang W, Sun L, Huo L, Zhao H (2019). Interface electronic coupling in hierarchical FeLDH(FeCo)/Co(OH)_2_ arrays for efficient electrocatalytic oxygen evolution. Chemsuschem.

[142] Zhang T, Hang L, Sun Y, Men D, Li X, Wen L, Lyu X, Li Y (2019). Hierarchical hetero-Ni_3_Se_4_@NiFe LDH micro/nanosheets as efficient bifunctional electrocatalysts with superior stability for overall water splitting. Nanoscale Horizons.

[143] Liu J, Wang J, Zhang B, Ruan Y, Lv L (2017). Hierarchical NiCo_2_S_4_@NiFe ldh heterostructures supported on nickel foam for enhanced overall-water-splitting activity. ACS Appl. Mater. Interfaces.

[144] Yang L, Xie L, Ge R, Kong R, Liu Z (2017). Core–shell NiFe-LDH@NiFe-Bi nanoarray: in situ electrochemical surface derivation preparation toward efficient water oxidation electrocatalysis in near-neutral media. ACS Appl. Mater. Interfaces.

